# The dynamin-related protein *Osdrp1c* plays an essential role in rice root elongation (*Oryza sativa* L.)

**DOI:** 10.3389/fpls.2026.1783261

**Published:** 2026-04-02

**Authors:** Yunyan Hua, Zhiqiang Guo, Lihuiying Jia, Wenwei Mu, Xu Li, Qiuping Li, Tao Ma, Yujie Chen, Shiyou Qiu, Weimei Zhang, Wona Ding

**Affiliations:** 1 Ningbo Key Laboratory of Agricultural Germplasm Resources Mining and Environmental Regulation, College of Science and Technology, Ningbo University, Ningbo, China; 2 Cixi Modern Agricultural Development Zone Management Center, Ningbo, China; 3 Lishui Institute of Agricultural and Forestry Sciences, Lishui, China

**Keywords:** DRP1C, *Oryza sativa*, reactive oxygen species, root architecture, ROS

## Abstract

Root is indispensable for anchoring, mechanical support, nutrient and water uptake, which is essential for plant growth and crop yield. However, the knowledge is largely unknown about the underlying molecular mechanisms that determine root growth and development. In this study, a rice (*Oryza sativa*) short root mutant named *Osdrp1c* which caused by reduced cell elongation, division and endocytic activity was obtained by using ethyl-methane sulfonate-mutagenized method. Moreover, the *Osdrp1c* mutant roots exhibited enhanced cell death and increased reactive oxygen species (ROS) accumulation. The mutation was mapped to and located in the putative *DRP1C* gene, as multidomain GTPases, dynamin-related proteins (DRPs) constitute a subgroup within the dynamin superfamily. OsDRP1C exhibited widespread expression across various plant tissues and was localized at chloroplast, cytoplasm and plasma membrane. In addition, transcriptome analysis showed that OsDRP1C modulates pathways for maintaining redox homeostasis like glutathione (GSH) metabolism and phenylpropanoid biosynthesis. Subsequent metabolomics further identified significant perturbations in related metabolic fluxes, impacting GSH, amino acid, nucleotide and phenylpropanoid metabolism. Therefore, Our results demonstrate that OsDRP1C orchestrates rice root development through its regulatory roles in dynamin-mediated cellular processes.

## 1 Introduction

The growth and development of plant roots constitute a complex biological process involving numerous genes and precisely regulated by endogenous hormonal signals and exogenous environmental stimuli. In recent years, with the advancement of functional genomics and molecular biology techniques, a large number of key genes controlling root initiation, morphogenesis, and environmental adaptation have been identified and cloned (Qin et al., 2022; Justamante et al., 2025). Based on their functions, these genes can be broadly categorized as follows: First, in responding to external nutrient and stress signals, genes such as the nitrate transporter gene *NRT1.1* not only participate in nitrogen uptake but also act as a nitrate sensor to regulate root foraging behavior (Justamante et al., 2025); meanwhile, the *DRO1* gene controls root growth angle, enabling roots to grow into deeper soil layers to access water under drought conditions (Justamante et al., 2025). Second, within the internal regulatory network of root development, auxin signaling pathway genes (e.g., *AUX1*, *PIN2*, and *TIR1*) play a central role in regulating root gravitropism and lateral root formation (Justamante et al., 2025). In cereal crops, the *WOX11* gene, which controls adventitious root initiation, and the ‘Green Revolution’ gene *Rht-D1b*, which regulates root apical meristem activity, have both been confirmed as key factors shaping root system architecture (Qin et al., 2022; Wang et al., 2025). Furthermore, under stress conditions like salinity, the role of transcription factors (e.g., MYB, NAC families) in activating suberin biosynthesis genes (e.g., *CYP86A1*) to establish an ion barrier has also been a recent research focus (Tariq et al., 2026). These discoveries collectively reveal the molecular basis of root plasticity and provide important genetic resources for crop genetic improvement.

Dynamin-related proteins (DRPs) are multidomain, mechanochemical GTPases that self-assemble and orchestrate a wide array of cellular processes (Ford and Chappie, 2019). They are classified into sub-families by sequence and functional domains and have been linked to cell plate formation, endocytosis, exocytosis, protein sorting, and division of mitochondria and chloroplasts (Collings et al., 2008). In addition, DRPs act in a variety of biological processes and contexts including clathrin-mediated endocytosis (dynamin), mitochondrial and peroxisomal fission (Drp1 and Dnm1), fusion (OPA1, Mgm1, and mitofusins), vacuolar dynamics and endosome regulation (Vps1), interferon-induced viral restriction (Mx), plant cell cytokinesis, membrane fission (*Arabidopsis* DRPs) and membrane binding and tethering (bacterial dynamin-like proteins) (Praefcke and McMahon, 2004; Hoppins et al., 2007; Antonny et al., 2016; Jimah and Hinshaw, 2019).

Dynamin and DRPs have been shown to play essential roles in cell division in plants (Kang et al., 2003a), animals (Thompson et al., 2002; Skop et al., 2004), and the protest (Wienke et al., 1999). Both *in vivo* and *in vitro* evidence indicates that animal dynamins, as DRP family members, act by assembling into rings or helices at the bud neck and subsequently severing the donor membrane in a GTP-dependent manner (Fujimoto and Tsutsumi, 2014). While DRP-mediated trafficking has been extensively studied in animal cells, its mechanisms and functions in plant cells remain largely elusive.

The first plant DRPs sequence (*DRP1*) was cloned and identified in *Arabidopsis thaliana* in 1995 (Dombrowski and Raikhel, 1995), and the first plant DRPs protein (phragmoplastin) was purified from soybean in the following year (Gu and Verma, 1996). Starting from these two results, plant DRPs has been significant progress in research. Currently, research on plant DRPs is mainly focused on *Arabidopsis*. In *Arabidopsis*, the 16 DRP family members are divided into six subfamilies (DRP1–6) according to phylogeny and protein motif composition. Each member within a subfamily is denoted by an additional letter suffix. The *Arabidopsis* DRP1 subfamily comprises five members (DRP1A–DRP1E) with high amino acid sequence identity (63–82%) (Fujimoto and Tsutsumi, 2014). DRP1-mediated membrane recycling is crucial for plasma membrane formation and maintenance in plants, primarily through the functionally redundant isoforms AtDRP1A and AtDRP1E. Consistent with this role, *atdrp1A*/*atdrp1E* double mutants exhibit defects in cell plate assembly, cell wall formation, and plasma membrane recycling, highlighting their essential function in polar cell expansion and cytokinesis (Kang et al., 2001, 2003). The defects in plasma membrane and intine morphology observed in *Atdrp1C* pollen during microspore maturation indicate that AtDRP1C is essential for forming and maintaining the pollen cell surface, thereby ensuring viability during desiccation (Kang et al., 2003b). Furthermore, AtDRP2 function is essential for plant growth. AtDRP2A and AtDRP2B function coordinately in multiple pathways of post-Golgi trafficking in phosphatidylinositol 3- or 4-kinase and cytoskeleton polymerization-dependent manner (Taylor, 2011; Huang et al., 2015). AtDRP3A and AtDRP3B are shared by peroxisomal and mitochondrial division, whereas the structurally-distinct AtDRP5B protein is involved in the division of chloroplasts and peroxisomes (Aung and Hu, 2012; Wang et al., 2012). Unfortunately, the functions of AtDRP4 and AtDRP6 are less studied. The AtDRP4 subfamily contains orthologues of the animal antiviral Mx proteins. AtDRP4A, AtDRP4B and AtDRP4D appear to be pseudogenes, and may have arisen recently during evolution and have yet to gain any function. Likewise, AtDRP6 is classified in the subfamily of unknown dynamin-related genes. It is not known if AtDRP6 gene is transcribed, and if its encoded protein is a GTPase (Hong et al., 2003).

In rice, the related studies of DRPs are only DRP1, DRP2 and DRP3. The rice dynamin-related protein 1E (OsDRP1E) negatively regulates programmed cell death (PCD) by controlling mitochondrial structure and cytochrome C release (Li et al., 2017). The OsDRP1E mutant *dj-lm* exhibited leaf surface disease-like spot traits, with the spot first appearing at the rice leaf tips and then expanding toward the whole leaf. Beyond displaying cell death and senescence phenotypes, the *dj-lm* plants also exhibited alterations in a spectrum of major agronomic traits, including plant height, seed setting rate, tiller number, flag leaf angle, 1000-grain weight, and panicle length (Li et al., 2017). BC3 (OsDRP2B) is tightly involved in the synthesis of cellulose and is essential for proper secondary cell wall construction (Hirano et al., 2010; Li et al., 2010; Xiong et al., 2010) Mutation of *OsDRP2B* disturbs the membrane trafficking that is essential for normal cellulose biosynthesis of the secondary cell wall, thereby leading to inferior mechanical properties in rice plants (Xiong et al., 2010). The *bc3* mutation specifically reduced cellulose content by 28–36% in culms, leaves, and roots, with no effect on other cell wall components (Hirano et al., 2010). The rice dynamin-like protein OsDRP3A is a homologue of *Arabidopsis* DRP3A and DRP3B, implicating it in the process of mitochondrial fission. *OsDRP3A* gene mutant *k63a* exhibited reduced number, larger size, and distorted morphology of mitochondria (Fujimoto et al., 2004). Except for the above 3 types of DRPs genes, there are no reports on the remaining DRPs. The study of rice DRPs is biologically important for us to explore their regulation of the growth and development of the rice organism.

Root system architecture is highly dynamic and plastic, meticulously regulated by an intricate interplay of internal genetic programs and external environmental cues (Su et al., 2024). For a long time, ROS were primarily viewed as harmful by-products of aerobic metabolism, capable of causing cellular oxidative damage. However, research over the past two decades has fundamentally revolutionized this view, establishing ROS—particularly hydrogen peroxide (H_2_O_2_)—as a crucial class of signaling molecules that play central roles in regulating a multitude of plant physiological processes (Su et al., 2024; Fedoreyeva and Kononenko, 2025). During root development, ROS signaling exhibits precise spatiotemporal distribution patterns. For instance, superoxide anions (O_2_^-^) predominantly accumulate in the root apical meristem zone, while H_2_O_2_ is enriched in the differentiation zone. This differential distribution is directly linked to distinct cell fate decisions—proliferation versus differentiation (Fedoreyeva and Kononenko, 2025). The “dual role” (dose-dependent) and “spatiotemporal specificity” of ROS form the foundation of its signaling function: ROS at low concentrations and specific locations participates in normal developmental signaling, whereas high-level accumulation leads to oxidative stress (Su et al., 2024; Fedoreyeva and Kononenko, 2025). The generation, homeostasis, and perception of ROS signals are governed by a sophisticated network. Plasma membrane-localized NADPH oxidases (RBOHs) are key enzymes responsible for producing signaling ROS, and their activity is finely regulated by mechanisms such as calcium ions and phosphorylation (Su et al., 2024; Gerber et al., 2025). Concurrently, antioxidant systems centered around GSH and ascorbate are responsible for scavenging excess ROS, maintaining redox homeostasis (Fedoreyeva and Kononenko, 2025). These components collectively constitute a responsive “redox signaling system,” allowing ROS to act as secondary messengers. By oxidatively modifying specific cysteine residues on target proteins, ROS rapidly alters protein activity, localization, or interactions, thereby transmitting signals in regulating root growth and development (Fedoreyeva and Kononenko, 2025; Gerber et al., 2025).

A novel rice mutant with short roots, named *Osdrp1c*, was isolated and characterized in this study. This mutant displays impairments in both root growth and major agronomic traits. Subsequent cloning identified the causal gene as encoding a rice dynamin, OsDRP1C. This protein shows constitutive expression in all examined tissues and stages and is localized at plasma membrane. To investigate how this dynamin regulates root development, we performed integrated transcriptomic and metabolomic profiling.

## 2 Materials and methods

### 2.1 Plant materials and growth conditions

The *Osdrp1c* mutant was isolated from an EMS-mutagenized population of rice (*O. sativa* L. *indica* cv. Kasalath), which was kindly provided by Prof. Ping Wu (Zhejiang University, China). *Osdrp1c-1*, *Osdrp1c-9*, *Osdrp1c-15* mutants in the Zhonghua 11 background were generated using CRISPR-Cas9 editing. For hydroponic culture, rice nutrient solution was adjusted to 5.5 (Zhu et al., 2012), rice plants were grown in a greenhouse maintained at 30°C/22°C with a 12/12 h light/dark cycle, and ~70% humidity. For comparative phenotyping of key agronomic traits, WT and mutant plants were cultivated side-by-side in a paddy field under natural environmental conditions. *Nicotiana benthamiana* plants were maintained in a climate chamber at 26°C and 60% humidity with a 16-h light (130 µmol m^-2^ s^-1^)/8-h dark cycle, using premixed soil.

### 2.2 Histological observation

Histological sections were prepared and examined following the protocol of Ding et al. (2018).

### 2.3 Acetocarmine and EdU staining

Procedures for acetocarmine and EdU staining was conducted with roots of 5-day-old WT and *Osdrp1c* plants as previously described (Ding et al., 2018). For each genotype, at least ten root samples were examined, with the entire experiment being conducted twice (n ≥ 10 per experiment).

### 2.4 FM4–64 uptake assay in rice toots and rice roots protoplast

Endocytosis was assayed by treating 7-day-old seedlings (grown in rice nutrient solution) with 10 μM FM4-64 (prepared in ddH_2_O) for 30 min at room temperature. Subsequently, root tips or root protoplasts were harvested and visualized via confocal laser scanning microscopy.

### 2.5 Cell death and ROS detection, hydroponic experiments treatment with chemical inhibitors

Cell death was assessed by Evans Blue staining. This dye selectively accumulates in dead cells due to compromised membrane integrity (Gaff and Okong'O-Ogola, 1971). Tissues were immersed in a 1 mg/mL aqueous Evans Blue solution for 10 minutes, briefly rinsed in distilled water, and subsequently imaged under a Nikon 90i microscope. *In situ* H_2_O_2_ production was detected using 3,3-diaminobenzidine (DAB) staining (1 mg/mL in ddH_2_O, 10 min) (Veljovic-Jovanovic et al., 2002). O_2_^-^ production was evaluated by Nitroblue tetrazolium chloride (NBT) staining according to Carol et al. (2005), using a 2 mM NBT solution in 20 mM phosphate buffer (pH 6.1) for a 10-minute incubation. Each experiment, involving more than ten tissue samples per genotype, was independently repeated two times.

A bought concentrated stock solution of H_2_O_2_ is 500 mM, this stock solution was then diluted with normal rice culture solution to provide a final working concentration of 10 μM and 50 μM for hydroponic experiments. Dimethythiourea (DMTU) (Jiang and Zhang, 2002; Li et al., 2016), a scavenger of H_2_O_2_, was used. A 150 mM stock solution (0.1563 g in 10 mL ddH_2_O) was prepared and then diluted with rice culture solution to final concentrations of 1 mM and 5 mM. The RBOH inhibitor Diphenyleneiodonium chloride (DPI) (Zhang et al., 2020) was applied at 0.01 μM. A 10 mM DPI stock (10 mg in 3.179 mL anhydrous DMSO) was diluted in rice culture solution to obtain the working concentration.

### 2.6 Pollen activity staining assay

Pollen viability was assessed by I_2_-KI staining. Spikelets at the pre-anthesis stage were collected from both WT and *Osdrp1c* plants. The hulls were carefully dissected, and anthers were transferred onto a glass slide containing a drop of 1% I_2_-KI solution. Anthers were then gently crushed to release pollen grains, and staining was observed under a light microscope (Nikon Eclipse 80i). For each genotype, samples from more than five individual plants were analyzed across two independent experiments.

### 2.7 Mapping and cloning of *OsDRP1C*

To map the *OsDRP1C* locus, we generated a mapping population by crossing the homozygous *Osdrp1c* mutant (*indica*) with the *japonica* cultivar Nipponbare. Primary mapping was conducted using 30 short-root F_2_ plants, while fine mapping employed 1112 such plants. This effort delimited the locus to a 131.5-kb interval between markers In2 and In3 on chromosome 3. Among the 19 predicted protein-coding genes in this region, whole-genome resequencing was performed on the target region. Candidate genes derived from the sequencing were analyzed through functional annotation and literature review. Furthermore, the mRNA from the mutant allele was cloned and sequenced, and the impact of the mutation on protein synthesis was evaluated via bioinformatic alignment analysis. Ultimately, *OsDRP1C* was identified as the candidate. We then amplified and sequenced both genomic DNA and cDNA of *OsDRP1C* from wild-type and mutant plants. All primers used are listed in **Supplementary Table 1**.

### 2.8 Construction of vectors and plant transformation

The *OsDRP1C* coding sequence, verified by cloning into pEASY^®^-Blunt Simple and sequencing, was assembled with CaMV35S promoter in the binary vector pCAMBIA1300 for complementation. A parallel GUS reporter construct (*OsDRP1C p::GUS*) was generated by fusing the same promoter to the *GUS* gene in pCAMBIA1300NH-GUS. These constructs were used for *Agrobacterium*-mediated transformation of rice (Chen et al., 2003). See **Supplementary Table 1** for primers.

### 2.9 Histochemical analysis and GUS assay

GUS activity was assessed histochemically according to Ding et al. (2015), with more than five samples examined per genotype or tissue type in two experimental replicates.

### 2.10 Subcellular localization of OsDRP1C

We constructed an OsDRP1C-GFP fusion by fusing the *OsDRP1C* ORF (stop codon removed) to the 5’ end of smGFP4. The fusion gene was cloned into the 35S-pCAMBIA1301 vector. The resulting construct was sequenced to verify in-frame fusion and used for transient transformation of rice protoplasts. Red fluorescence signal of chloroplast auto-fluorescence as a marker. GFP and RFP fluorescence was visualized with a Zeiss LSM 510 confocal microscope, with the experiment repeated twice to ensure reproducibility.

### 2.11 RNA extraction, reverse transcription and RT-qPCR

Gene expression was analyzed by RT-qPCR on RNA extracted from roots and leaves of 7-day-old WT and *Osdrp1c* plants (three biological replicates). cDNA was synthesized using HiScript II RTase, and qPCR was performed on an ABI 7500 system. Data were analyzed by the 2^(-ΔΔCT) method (Livak and Schmittgen, 2001) with *OsActin* as the control (primers in **Supplementary Table 1**). PCR products were checked on agarose gels.

### 2.12 Transcriptome sequencing and differentially expressed gene analysis

Root RNA from 7-day-old WT and *Osdrp1c* seedlings (three biological replicates per genotype) was extracted using a Qiagen RNeasy kit for transcriptome sequencing and differential expression analysis, as previously described (Ye et al., 2020). The transcriptome sequencing and analysis were conducted by OE biotech Co., Ltd. (Shanghai, China).

### 2.13 Untargeted metabolomic profiling

For metabolomic profiling, metabolites were extracted from roots of WT and Osdrp1c rice using a methanol/chloroform/water system with 2-chloro-L-phenylalanine as an internal standard. After extraction, homogenization, and centrifugation, the supernatants were lyophilized and derivatized sequentially with methoxyamine hydrochloride and BSTFA. Derivatized samples were analyzed by GC-MS (Agilent 7890B/5977A). Multivariate data and pathway analyses were conducted in MetaboAnalyst 4.0 software (http://www.metaboanalyst.ca/). The untargeted metabolomic profiling and analysis were conducted by Lu-Ming Biotech Co.,Ltd. (Shanghai, China).

## 3 Results

### 3.1 Rice *Osdrp1c* is a mutant with short root, shoot, and reduced endocytic activity

**Figure 1:**
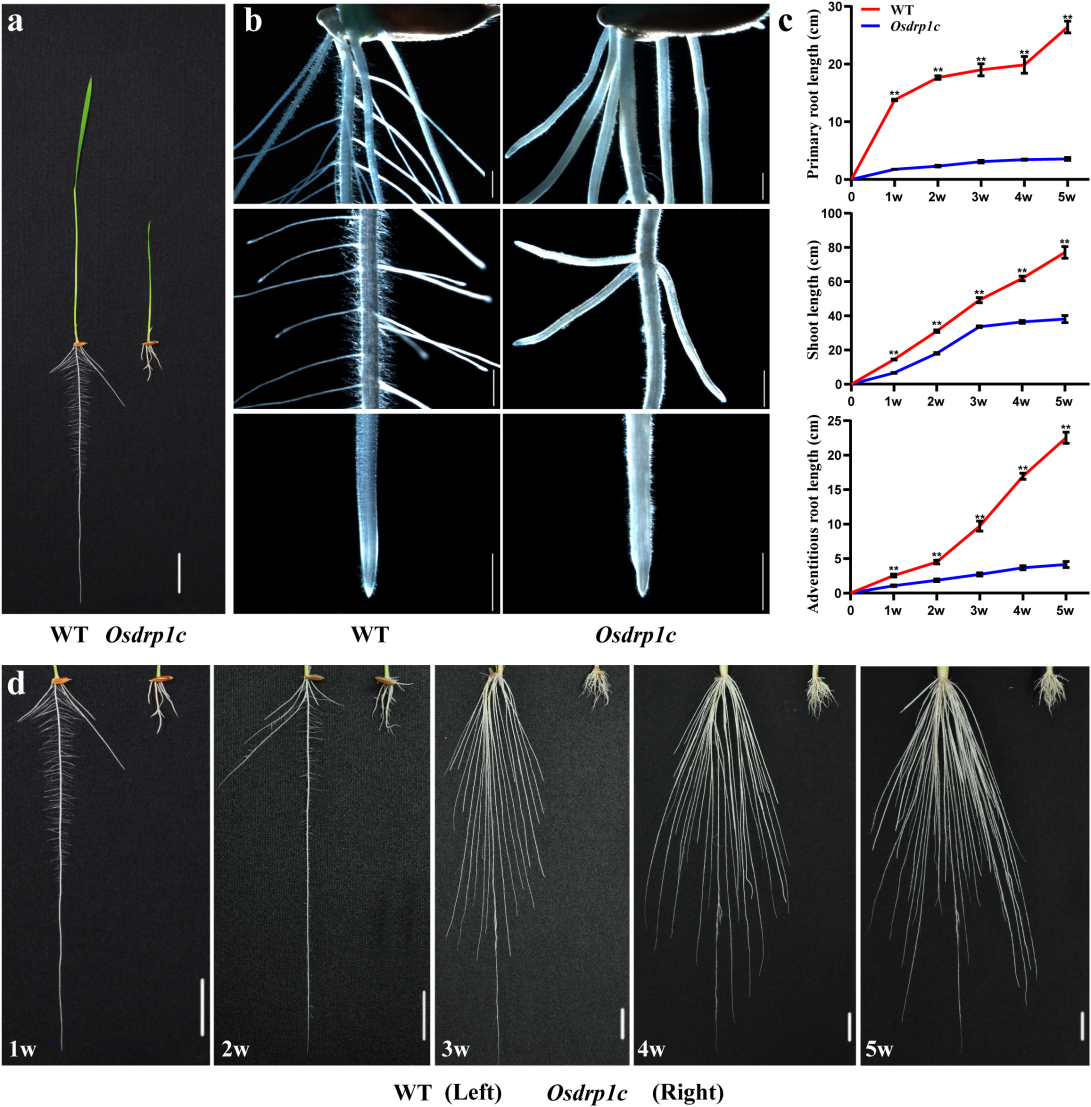
Phenotypic characterization of the WT (Kas) and *Osdrp1c* (EMS-mutagenized *Oryza sativa L. indica* cv Kasalath). **(a)** Growth of 7-day-old seedings of the WT (left) and *Osdrp1c* (right). **(b)** Stereomicroscope images of roots of the WT (left) and *Osdrp1c* (right). **(c, d)** Quantification of primary roots, adventitious roots, and lateral roots in WT and *Osdrp1c* from 1 week to 5 weeks **(c)** and phenotypic comparison **(d)**, w, week. Significant differences were determined using Student’s test (**P < 0.01). Bar 2 cm **(a, d)**, 1 mm **(b)**.

From an EMS-mutagenized population of rice (*Oryza sativa* L. *indica* cv. Kasalath), we isolated a short-root mutant, which we named *Osdrp1c* based on subsequent gene characterization. At the early seedling stage, *Osdrp1c* displayed significantly retarded growth in both roots and shoots (**Figure 1a**). The *Osdrp1c* mutant exhibited severe impairments in the development of its primary, adventitious, and lateral roots compared to the wild type (**Figure 1b**). Following 7 days of growth in normal rice culture solution, the primary root length of mutant seedlings was dramatically reduced to 13.73% of that observed in WT plants (**Figure 1a**). *Osdrp1c* also displayed a milder short shoot phenotype compared to the WT (**Figure 1a**). Longitudinal observation revealed an increasingly severe short root/shoot phenotype in *Osdrp1c* during development, with a concomitant recovery in adventitious root number (**Figures 1c, d**, **S1**).

**Figure 2:**
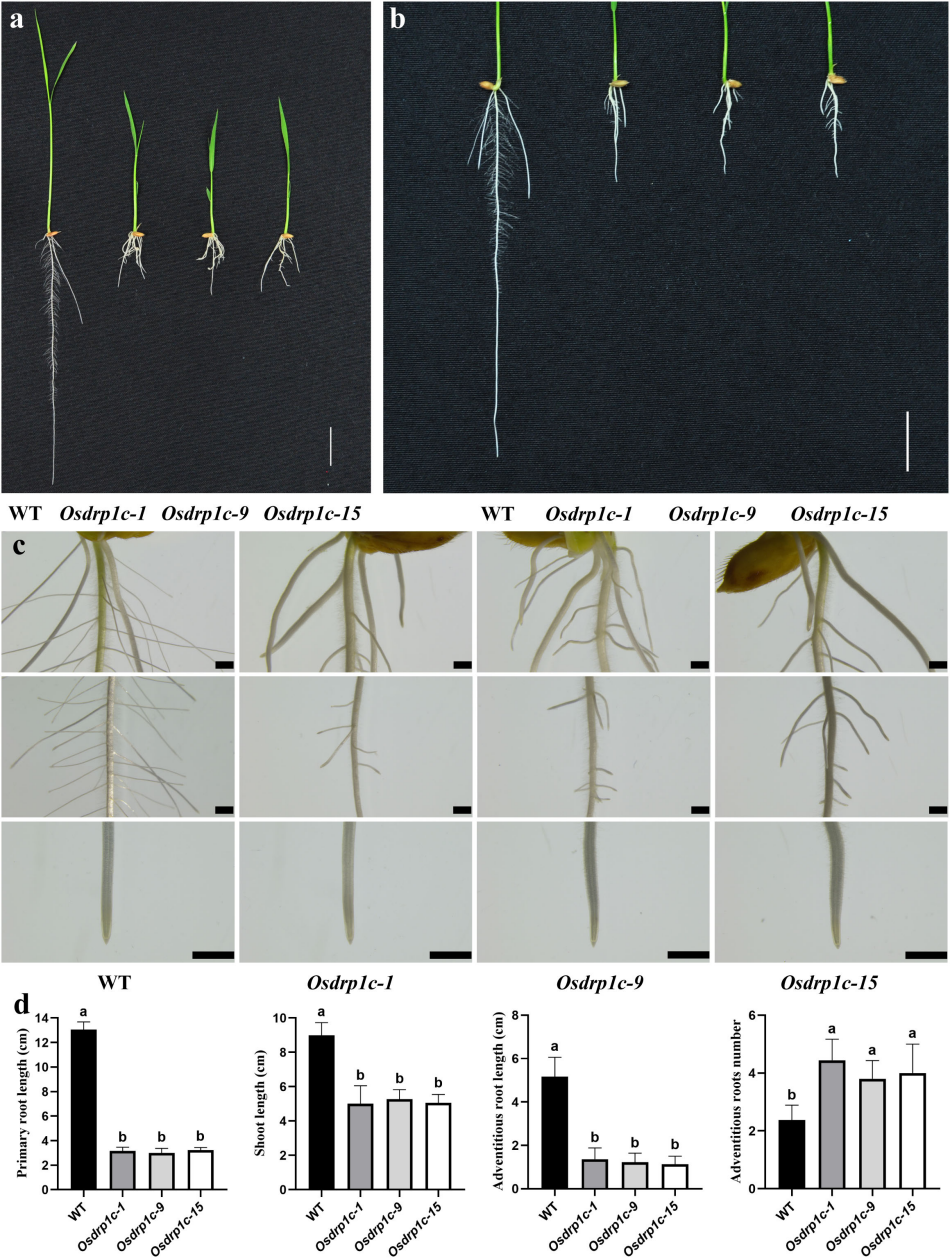
Phenotypic characterization of the WT (ZH11) and 3 mutant lines of *Osdrp1c* (-1, -9, -15) (CRISPR-Cas9 mutagenized *Oryza sativa* L. *japonica* var. Zhonghua 11). **(a)** Growth of 7-day-old seedings of the WT (ZH11) and *Osdrp1c* 3 mutant lines. **(b)** 7-day-old seedings root phenotypes of WT (ZH11) and *Osdrp1c* 3 mutant lines. **(c)** Stereomicroscope images of roots of the WT (left) and *Osdrp1c* 3 mutant lines (right). **(d)** Quantification of shoot, primary roots, adventitious roots in WT (ZH11) and *Osdrp1c* 3 mutant lines which cultivated for 1 week. Different letters indicate significant differences according to Tukey’s multiple comparisons test. Columns with different marked letters are significantly different (*P < 0.05). Bar 2 cm **(a, b)**, 1 mm **(c)**.

To further confirm the role of *OsDRP1C* in rice root development, *Osdrp1c* mutants were generated using CRISPR-Cas9 editing. Three mutant lines, *Osdrp1c* (-1, -9, -15), with frameshift mutations at position 56 which alters all downstream amino acids were obtained for detailed analysis (**Supplementary Figure 2**). As shown in **Figure 2**, both *Osdrp1c* (-1, -9, -15) exhibited shorter roots and shoots than WT (ZH11). The short root phenotype of *Osdrp1c* (-1, -9, -15) are consistent with EMS-mutagenized rice line *Osdrp1c* showed in **Figure 1**.

**Figure 3:**
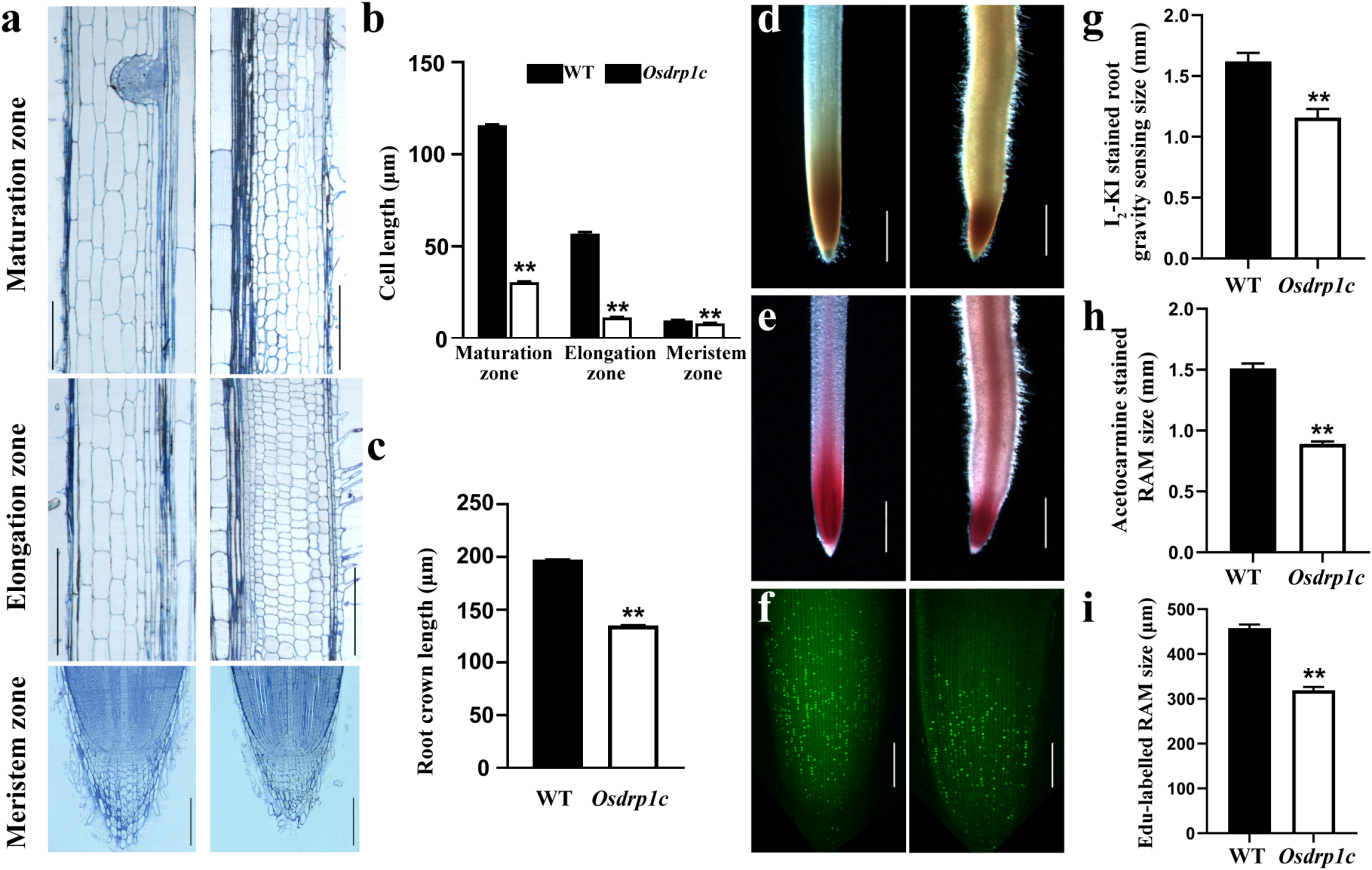
Longitudinal sections of the root maturation zone (top), elongation zone (middle) and meristem zone (bottom) of 3-day-old WT and *Osdrp1c* plants and detection of the primary root gravity sensing activity and primary root meristem activity of 5-day-old WT and *Osdrp1c*. **(a)** Longitudinal sections of the maturation zone (top), elongation zone (middle) and meristem zone (bottom) of WT (left) and *Osdrp1c* (right). **(b, c)** Cell length **(b)** and root crown length **(c)** of WT and *Osdrp1c*.**(d)** I_2_-KI staining of root tips of WT (left) and *Osdrp1c* (right). **(e)** Acetocarmine staining of root tips of WT (left) and *Osdrp1c* (right). **(f)** S-phage entry of WT (left) and *Osdrp1c* (right) root tips visualized by EdU staining. **(g)** Size of I_2_-KI-stained root gravity sensing of WT and *Osdrp1c*. **(h)** Size of acetocarmine-stained root apical meristems (RAMs) of WT and *Osdrp1c*. **(i)** Size of EdU-labelled RAMs of WT and *Osdrp1c*. Significant differences were determined using Student’s test (**P < 0.01). Bar 100 μm **(a)**, 1 mm **(d, e)**, 100 μm **(f)**.

We examined longitudinal and cross sections of WT and *Osdrp1c* roots to determine the cellular basis of the short root phenotype (**Figures 3a**, **S3**). No significant differences were observed in the number of cell layers in the maturation, elongation, and meristematic zones between WT and *Osdrp1c* roots (**Supplementary Figure 3**). Measurements showed that cell length in the elongation and maturation zones of *Osdrp1c* was significantly reduced (**Figures 3a, b**), indicating a primary defect in cell elongation rather than division. Meanwhile, the root crown length of *Osdrp1c* was 68.37% shorter than WT (**Figure 3c**). However, *Osdrp1c* has wider cell width in the maturation and elongation zones than WT, but narrower in the meristematic zone (**Figure 3a**). We evaluated root meristem activity using EdU labeling and acetocarmine staining. EdU incorporation was significantly lower in *Osdrp1c* meristems (**Figures 3f, i**), and the mutant’s meristematic zone was visibly reduced (**Figures 3e, h**), jointly implicating defects in cell division and elongation. We also examined gravity sensing via I_2_-KI staining, which showed a shorter accumulation zone in the mutant root tip (**Figures 3d, g**).

**Figure 4:**
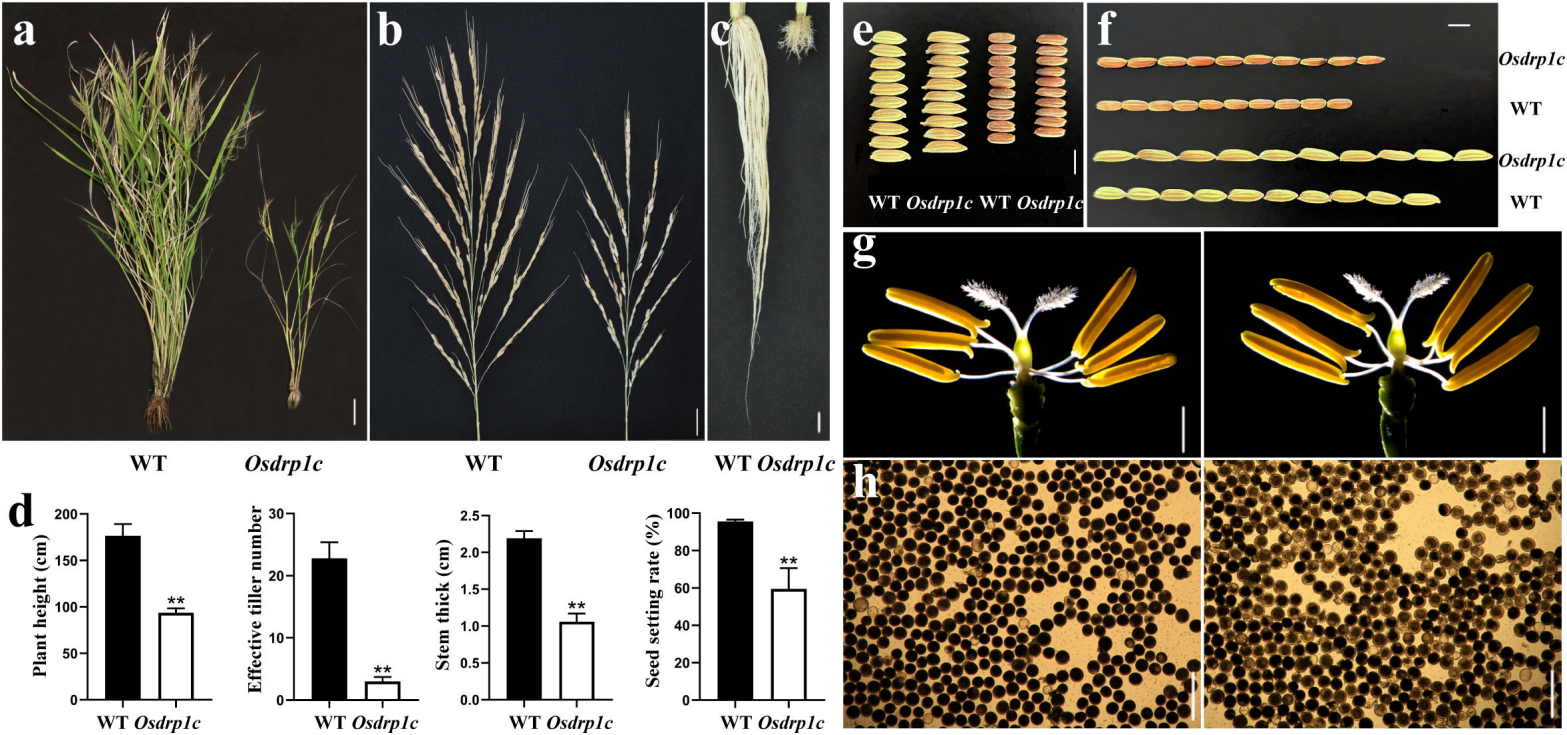
Comparison of agronomic traits and reproductive organs in the WT and *Osdrp1c* mutant at mature stage. **(a)** Mature WT (left) and *Osdrp1c* (right) plants. **(b)** Mature panicles of the WT (left) and *Osdrp1c* (right). **(c)** Roots of the WT (left) and *Osdrp1c* (right). **(d)** Plant height, effective tiller numbers, stem thick and seed setting rate in WT and *Osdrp1c*. Significant differences were determined using Student’s test (**P < 0.01). **(e)** Grain width of the WT and *Osdrp1c* (Rough rice (with husk) and brown rice (dehulled)). **(f)** Grain length of the WT and *Osdrp1c* (Rough rice (with husk) and brown rice (dehulled)). **(g)** Pistils and stamens of the WT (left) and *Osdrp1c* (right). **h** I_2_-KI solution staining of pollen from the WT (left) and *Osdrp1c* (right). Bar 10 cm **(a)**, 2 cm **(b, c)**, 4 mm **(e, f)**, 1 mm **(g)**, 100 μm **(h)**.

The developmental defects of *Osdrp1c* persisted into the mature stage, leading to significant reductions in plant height, effective tiller number, seed-setting rate, and stem thickness compared to the WT (**Figures 4a,b, d**). Nonetheless, the thousand-grain weight remained unchanged (data not shown). To investigate the underlying cause of the fertility defect, we examined reproductive organs. While pistil and stamen morphology appeared normal (**Figure 4g**), pollen development was dysfunctional, resulting in markedly lower fertility (**Figure 4h**). Interestingly, *Osdrp1c* produced grains that were both longer (6.00 ± 0.02 mm) and thicker (1.93 ± 0.01 mm) than those of the WT (5.3 mm*2.1 mm) (**Figures 4e, f**; **Supplementary Figure S4**).

**Figure 5:**
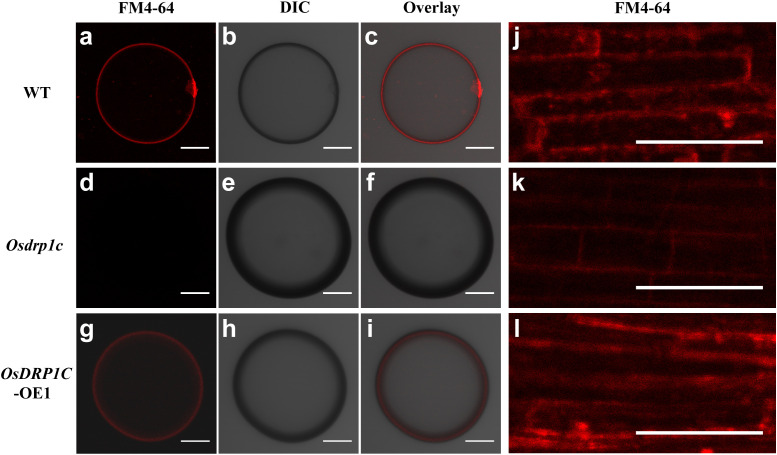
Analysis of endocytic activity in wild-type (WT) and the mutants. **(a–i)** Rice roots protoplasts stained with an endocytic tracer FM4–64 in WT **(a–c)**, *Osdrp1c* **(d–f)**, *OsDPR1C*-OE1 **(g–i)**. FM4-64 **(a, d, g)** signals, bright field **(b, e, h)** and the overlay images **(c, f, i)** are shown. **(j–l)** FM4–64 uptake in root tips in WT, *Osdrp1c* and *OsDRP1C*-OE1. Scale bars= 50 μm.

Preview study reported that dynamin-related protein positively correlated with endocytic activity (Kawai et al., 2022). To investigate whether *OsDRP1C* affect endocytic activity, fluorescent endocytic tracer FM4–64 was used to monitored endocytic activity in rice protoplast and root tips of WT, *Osdrp1c* and *OsDRP1C* overexpression line (*OsDRP1C*-OE1). The fluorescence signal intensity of *Osdrp1c* protoplast (**Figures 5a–i**) and root tips (**Figures 5j–l**) were weaker than that of WT and *OsDRP1C*-OE lines, which consist with preview study (Kawai et al., 2022).

To determine whether endocytic activity was altered, we performed FM4–64 uptake assays in roots of the WT, *Osdrp1c* mutant, and *OsDRP1C*-OE1 overexpression line. In *Osdrp1c*, the FM4–64 signal remained predominantly at the plasma membrane, and its intracellular uptake was diminished compared to the WT (**Figures 5j, k**), and this phenotype was recovered in *OsDRP1C*-OE1 (**Figures 5k, l**). Therefore, *OsDPR1C* does affect endocytic activity.

### 3.2 Elevated cell death alongside diminished ROS accumulation were exhibited in the root of *Osdrp1c*

**Figure 6:**
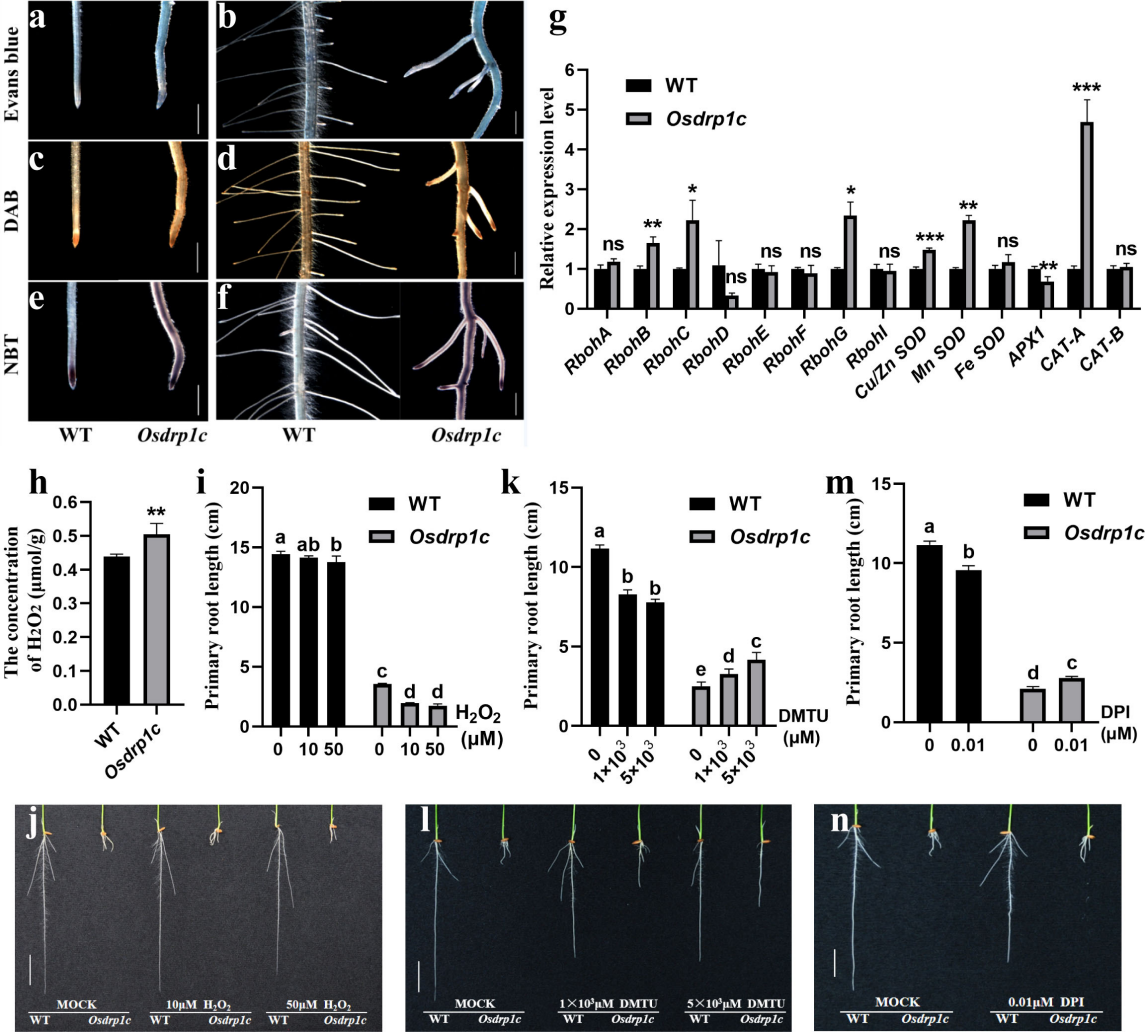
Detection of cell death and ROS accumulation levels of 7-day-old WT and *Osdrp1c*. **(a, b)** Evans blue staining of RAMs **(a)** and lateral root **(b)** of WT (left) and *Osdrp1c* (right). **(c, d)** DAB staining of RAMs **(c)** and lateral root **(d)** of WT (left) and *Osdrp1c* (right). **(e, f)** NBT staining of RAMs **(e)** and lateral root **(f)** of WT (left) and *Osdrp1c* (right). **(g)** Identification of transcriptional levels of genes in ROS production and scavenging pathways of 7-day-old WT and *Osdrp1c* roots. **(h)** H_2_O_2_ content increased in *Osdrp1c* plants. **(i, j)** Phenotype observation **(j)** and primary root length **(i)** of 7-day-old WT and *Osdrp1c* plants after being cheated with different concentration of H_2_O_2_. **(k, l)** Phenotype observation **(l)** and primary root length **(k)** of 4-day-old WT and *Osdrp1c* plants after being cheated with different concentration of DMTU. **(m, n)** Phenotype observation **(n)** and primary root length **(m)** of 4-day-old WT and *Osdrp1c* plants after being cheated with different concentration of DPI. Bars represent the standard errors of the means from three biological repeats. A two-sample unequal variance directional Student’s test was used to test the significance of the differences (*P < 0.05; **P < 0.01; ***P < 0.001). Different letters indicate significant differences according to Tukey’s multiple comparisons test. Columns with different marked letters are significantly different (*P < 0.05), and those with the same marked letter or share a letter are not significantly different. Bar 1 mm **(a–f)**, 2 cm **(j, l, n)**.

*Osdrp1c* roots displayed increased fragility compared to the WT. This observation was supported by Evans blue staining, which showed large areas of cell death at the root tips of the mutant, confirming compromised tissue viability (**Figures 6a, b**).

To assess whether ROS homeostasis was disrupted in *Osdrp1c*, we measured ROS levels in root tips of 7-day-old seedlings. O_2_^-^ was detected *in situ* by NBT staining, and H_2_O_2_ by DAB staining. Both assays revealed a significant accumulation of H_2_O_2_ (**Figures 6c, d**) and O_2_^-^ (**Figures 6e, f**) in *Osdrp1c* roots compared to the WT, particularly in lateral root tips and the central cylinder. These results indicate that OsDRP1C plays a critical role in ROS scavenging in roots.

Furthermore, we determined the content of H_2_O_2_ in *Osdrp1c* mutant plants and found that H_2_O_2_ content in the silenced plants was significantly increased 15.04% than controls (**Figure 6h**). We also determined the expression of genes involved in ROS production or scavenging by qRT-PCR in the roots of *Osdrp1c* and WT. Genes involved in O_2_^−^ production, such as members of the *RESPIRATORY BURST OXIDASE HOMOLOGS* (*RBOHs*) family (Guo et al., 2020), including *RbohB*, *RbohC*, and *RbohG*, were all significantly up-regulated (**Figure 6g**). Similarly, genes encoding Cu/Zn superoxide dismutase (Cu/Zn SOD) and Mn SOD, which are involved in H_2_O_2_ production, were also significantly up-regulated. Conversely, genes encoding APX1 (Guo et al., 2020), involved in H_2_O_2_ scavenging, were down-regulated, whereas genes encoding CAT-A (Xie et al., 2018), also involved in H_2_O_2_ scavenging, were up-regulated. The overall trend of transcriptional levels of genes in ROS production and scavenging pathways in the leaves of WT and *Osdrp1c* was consistent with that in the roots (**Supplementary Figure 5**). These results demonstrate that ROS accumulated in *Osdrp1c* plants.

To determine whether the accumulation of ROS plays a role against root elongation in *Osdrp1c*, we first treated *Osdrp1c* with different concentration of H_2_O_2_, which was added in normal rice culture solution, then examined its effect on WT and *Osdrp1c* roots elongation after germinated and cultured in this solution for 7 days (**Figures 6i, j**). After being cheated with H_2_O_2_, the roots of *Osdrp1c* were much shorter compared with MOCK control, indicating that the accumulated ROS in *Osdrp1c* plays a role against root elongation.

To further confirm this result, we next treated *Osdrp1c* with DMTU to eliminate existing ROS and DPI to suppress the production of ROS, it was expected that DMTU and DPI treatment would decrease the accumulation of ROS in *Osdrp1c* and its root length would partially recover. Indeed, compared with MOCK control, the roots of *Osdrp1c* were less O_2_^−^ (**Supplementary Figure 6**) and much longer after being cheated with DMTU (**Figures 6k, l**) and DPI (**Figures 6m, n**), besides, the roots of WT were much shorter after being cheated with DMTU (**Figures 6k, l**) and DPI (**Figures 6m, n**) than non-treated control. Taken together, these results further demonstrate that the accumulated ROS in *Osdrp1c* plays a role against root elongation.

### 3.3 Cloning of the *OsDRP1C* gene

**Figure 7:**
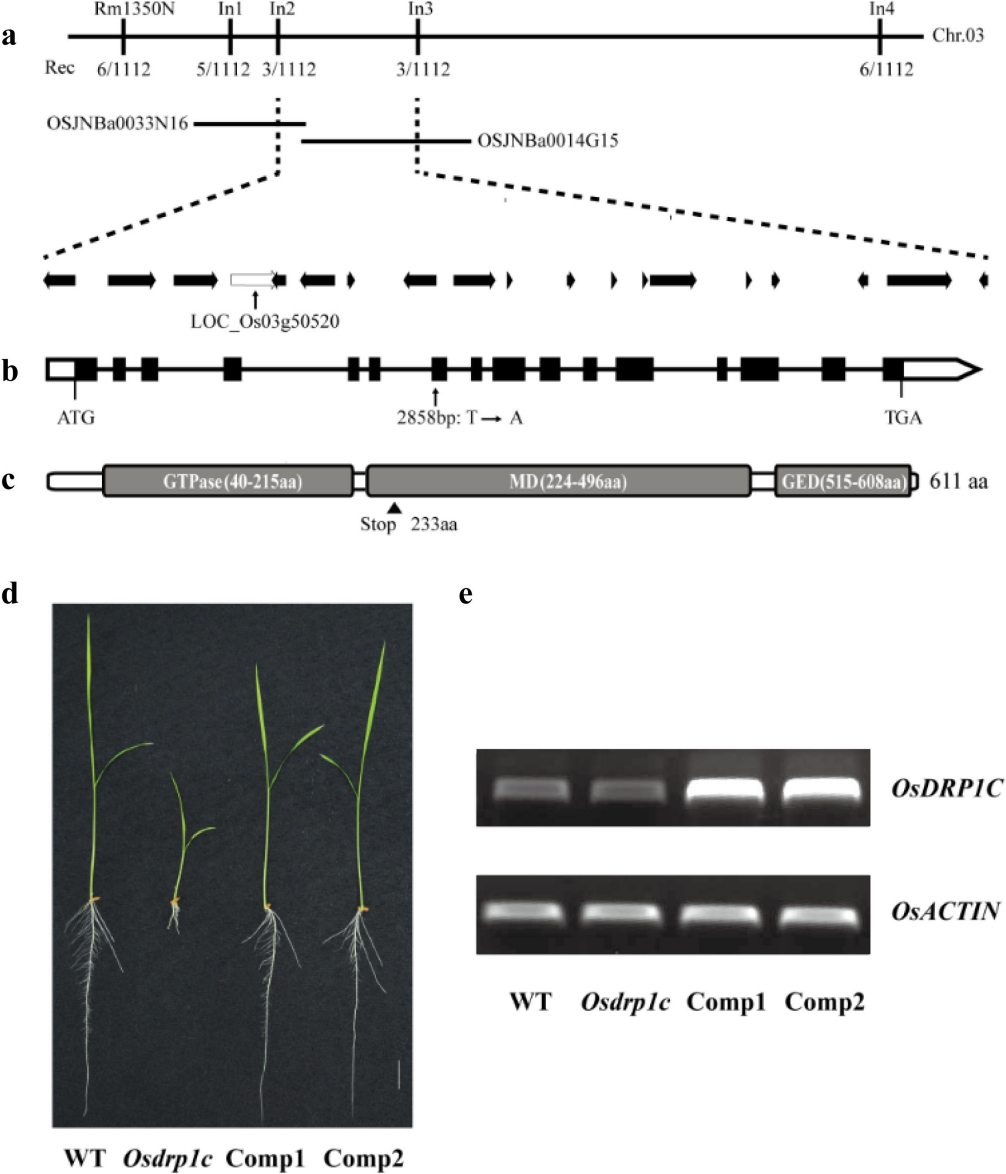
Map-based cloning of *OsDRP1C*. **(a)** Map-based cloning of *OsDRP1C* within a 131.5 kb region between InDel markers In2 and In3 on chromosome 3. Nineteen putative open reading frames were located in the 131.5 kb region. **(b)** The gene structure of *OsDRP1C*. Black boxes and lines represent exons and introns, respectively. White boxes indicate untranslated regions. The arrowhead shows the site of a single-base substitution (T to A) at the nucleotide 2858 bp downstream of ATG. **(c)** Predicted domains of *OsDRP1C* by the SMART database (https://smart.embl-heidelberg.de/). The arrowhead indicates the substitution within the MD domain, which leading to early termination of translation (Tyr^233^ to stop). **(d, e)** Complementation analysis of the *Osdrp1c* mutant. From left to right **(d)**: WT, *Osdrp1c*, and two independent lines of over-expression transgenic plants (Comp1 and Comp2) in the *Osdrp1c* mutant background. RT-PCR analysis **(e)** of *OsDRP1C* in roots of two independent over-expression transgenic lines. Bar 2 cm.

To map the causal mutation, we generated an F_2_ population from a cross between the *Osdrp1c* mutant and the *japonica* cultivar Nipponbare. The short-root phenotype segregated in a 3:1 ratio (WT:mutant = 379:131; χ²=2.89, *P* < 0.05), indicating control by a single recessive gene. Fine mapping with 1112 mutant seedlings delimited the locus to a 131.5-kb interval between markers In2 and In3 on chromosome 3 (**Figure 7a**). This region harbors 19 predicted open reading frames, including a dynamin-related protein 1C gene (*OsDRP1C*, LOC_Os03g50520), an ortholog of *Arabidopsis DRP1C* which regulates cell division and expansion (Kang et al., 2003a). Therefore, sequencing the *OsDRP1C* coding region in both WT and *Osdrp1c* revealed a point mutation (T to A) at position 2858 bp within the second exon (**Figure 7b**). This mutation introduces a premature stop codon (Tyr233 to stop) in a gene comprising 16 exons and encoding a 611-amino-acid protein (1836 bp CDS). Domain analysis via the SMART database identified canonical GTPase, middle (MD), and GTPase effector (GED) domains (**Figure 7c**).

For functional validation, the WT *OsDRP1C* coding sequence driven by the 35S promoter was introduced into *Osdrp1c* via the binary vector pCAMBIA1300. All examined transgenic lines (n>10) exhibited full restoration of root and shoot growth (**Figures 7d, e**, **S7**), confirming that the point mutation in *OsDRP1C* is responsible for the mutant phenotype.

### 3.4 Investigating the expression profile and subcellular localization of *OsDRP1C*

**Figure 8:**
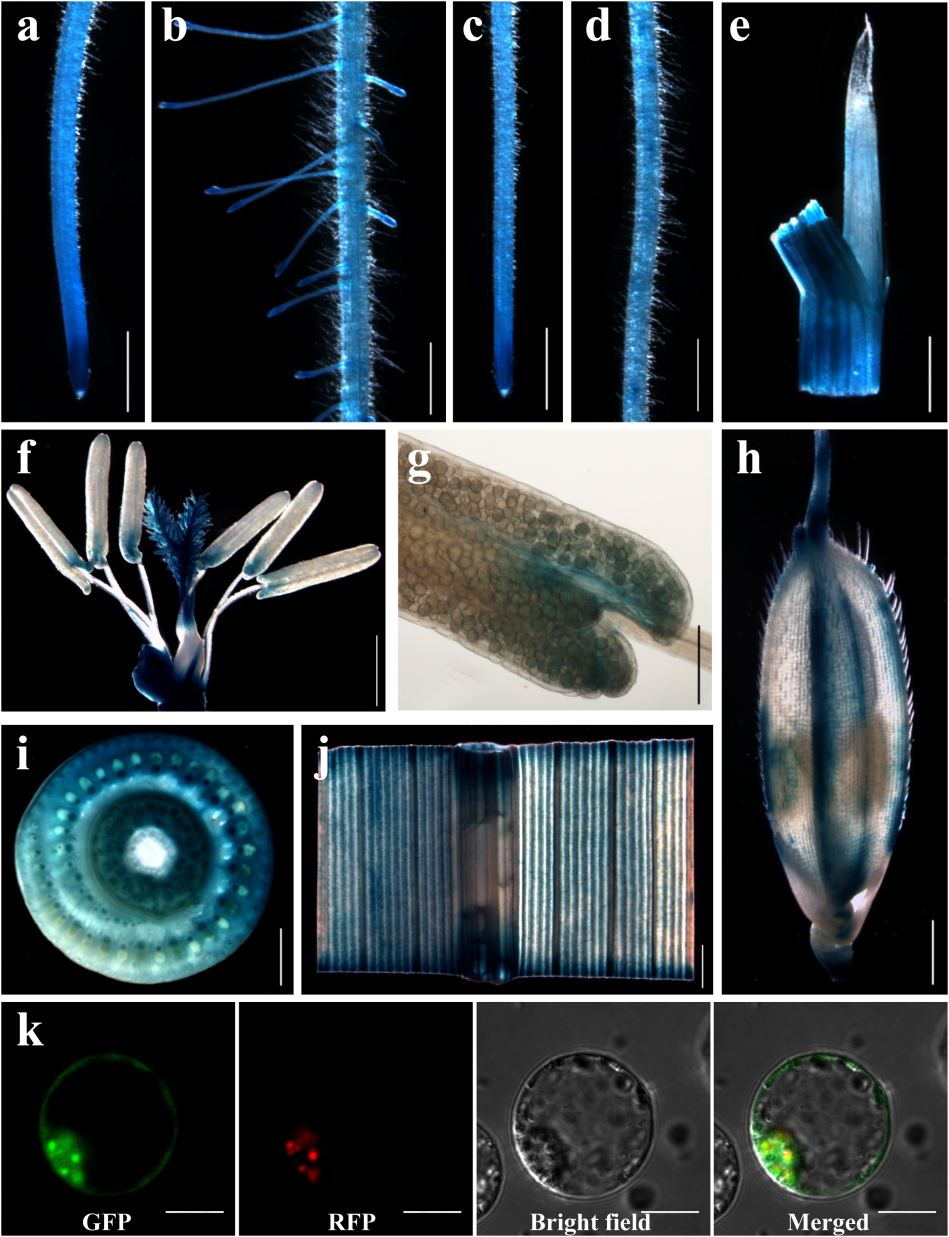
Expression pattern of OsDRP1C and subcellular localization of OsDRP1C. **(a–j)** Promoter-GUS fusion studies reveal the expression of OsDRP1C in various tissues, primary root tip **(a)**, primary root maturation zone **(b)**, adventitious root tip **(c)**, adventitious root maturation zone **(d)**, ligule **(e)**, flower **(f)**, stamen **(g)**, lemma **(h)**, the node region of stem **(i)**, leaf **(j)**. **(k)** OsDRP1C targets GFP to chloroplast in transiently transformed rice protoplasts cells. Bar 1 mm **(a-f, h-j)**, 100 μm **(g)**, 10 μm **(k)**.

To determine the tissue-specific expression of *OsDRP1C*, a 2.5-kb native promoter fragment was fused to the *GUS* reporter gene and introduced into rice via *Agrobacterium*-mediated transformation. Histochemical staining revealed ubiquitous GUS activity throughout the plant, including roots, leaves, pistils, stamens, glumes, and stem nodes (**Figures 8a-j**). Notably, strong expression was detected in the tips of primary, lateral, and adventitious roots (**Figures 8a-c**).

For subcellular localization, the *OsDRP1C* coding sequence was fused in-frame to *GFP* under the control of the 35S promoter. This construct was transiently expressed in rice protoplasts. Confocal microscopy showed that the OsDRP1C-GFP fluorescence signal co-localized with the the red fluorescence signal of chloroplast auto-fluorescence; besides, OsDRP1C-GFP fluorescence signal located at cytoplasm and plasma membrane (**Figure 8k**), indicating that OsDRP1C is a chloroplast and plasma membrane-localized protein.

### 3.5 Transcriptome analysis of *Osdrp1c*

To elucidate the molecular function of OsDRP1C, we performed RNA-seq transcriptome analysis on roots of 7-day-old wild-type and *Osdrp1c* seedlings (three biological replicates per genotype). Using a threshold of FDR < 0.05 and |fold change| > 1.5, we identified 8,882 differentially expressed genes (DEGs), of which 4,607 were up-regulated and 4,275 were down-regulated in the mutant (**Supplementary Figure 8a**).

**Figure 9:**
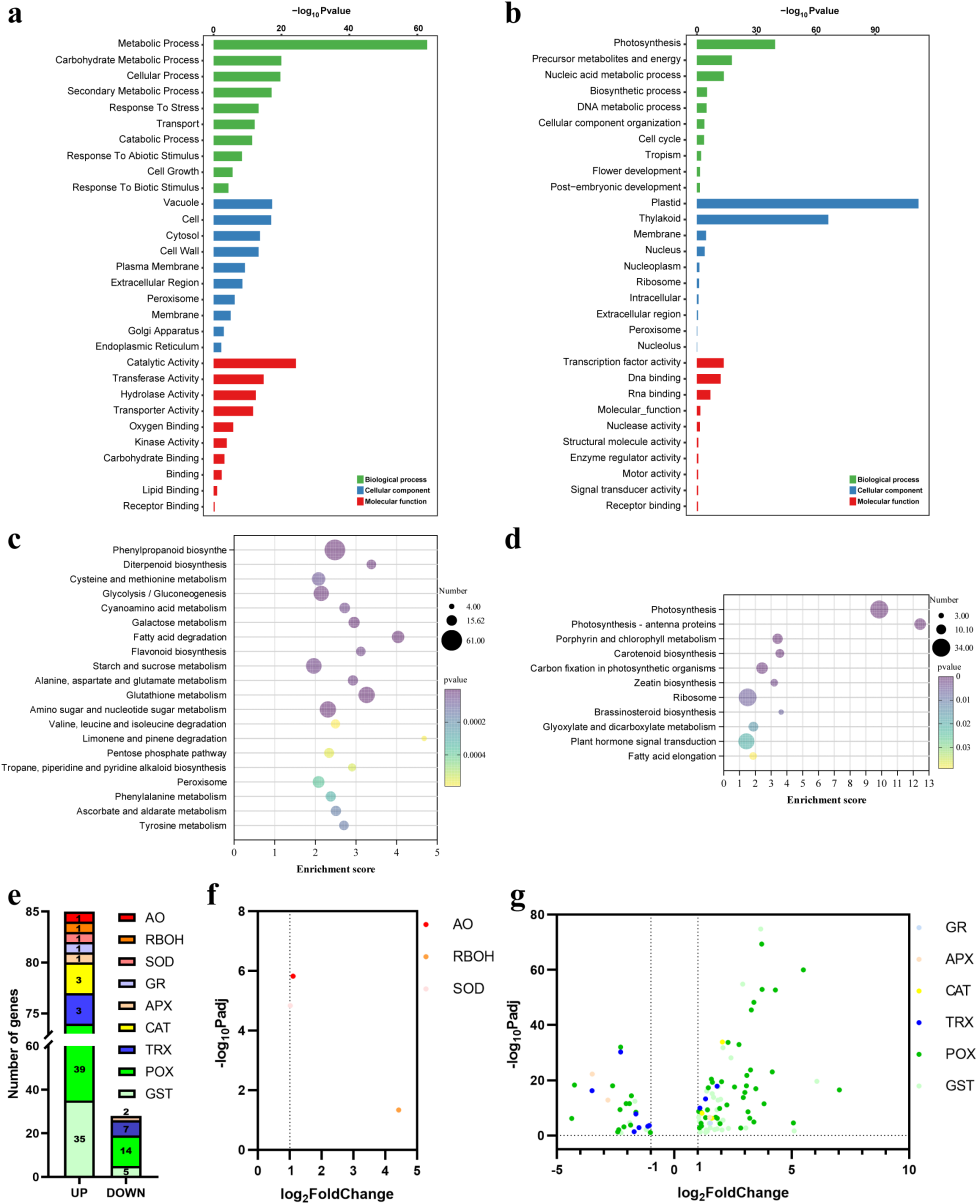
Transcriptomic analysis of roots of 7-day-old *Osdrp1c* by RNA-seq. **(a, b)** Gene ontology (GO) enrichment of up- **(a)** and down-regulated **(b)** differentially expressed genes (DEGs) between WT and *Osdrp1c*, respectively. **(c, d)** KEGG pathway enrichment of up- **(c)** and down-regulated **(d)** DEGs between WT and *Osdrp1c, P* < 0.05. **(e)** Number of genes from ascorbate oxidase (AO), respiratory burst oxidase homologues (RBOH), superoxide dismutase (SOD), glutathione reductase (GR), ascorbate peroxidase (APX), catalase (CAT), thioredoxin (TRX), peroxidase (POX) and glutathione-S transferase (GST) gene families, which displayed differentiation expression between WT and *Osdrp1c.* **(f)** Dynamic expression pattern of ROS production genes between WT and *Osdrp1c*. **(g)** Dynamic expression pattern of ROS scavenging genes between WT and *Osdrp1c*.Log_2_FoldChange ≥1.0, and ≤-1.0 with Padj<0.05 was considered as up-regulated and down-regulated, respectively and was plotted with -log_10_Padj in **(f–g)**. Different gene families are highlighted with distinct colors; UP, up-regulated; DOWN, down-regulated.

Gene Ontology (GO) enrichment analysis of these DEGs revealed distinct functional shifts. Up-regulated genes were primarily associated with stress responses, secondary metabolism, and general metabolic processes (**Figure 9a**), suggesting activation of defense pathways. In contrast, down-regulated genes were significantly enriched in processes related to photosynthesis, nucleic acid and DNA metabolism, biosynthesis, and cellular organization (**Figure 9b**), indicating a broad suppression of cell division and growth, consistent with the observed root developmental defects. At the molecular function level, catalytic activities (e.g., transferase, hydrolase) were enriched among up-regulated genes, while DNA/RNA binding and transcription factor activities were prominent among down-regulated genes. Cellular component analysis showed up-regulation linked to vacuole, cytosol, cell wall, and plasma membrane, whereas down-regulated genes were associated with plastid, thylakoid, membrane, nucleolus, and ribosome components.

To elucidate the pathways associated with OsDRP1C, we conducted KEGG enrichment analysis on the DEGs. A total of 43 pathways were enriched for up-regulated genes, and 11 for down-regulated genes. The up-regulated DEGs were predominantly clustered within the top 20 pathways related to: (i) carbohydrate metabolism (e.g., galactose, citrate cycle, glycolysis); (ii) lipid metabolism (e.g., fatty acid degradation); (iii) amino acid metabolism (e.g., valine and cysteine metabolism); and (iv) stress/defense processes such as phenylpropanoid biosynthesis, glutathione metabolism, and proteasome activity (**Figure 9c**). The down-regulated DEGs were enriched in pathways related to ribosome, photosynthesis (including antenna proteins, porphyrin/chlorophyll metabolism, carotenoid biosynthesis and carbon fixation) and plant hormone signal transduction (zeatin and brassinosteroid biosynthesis). These findings collectively indicate a broad suppression of biosynthetic activities for carbohydrates and proteins in *Osdrp1c* (**Figure 9d**).

Detailed inspection of the KEGG pathways revealed that all enzymatic steps in both the fatty acid degradation and phenylpropanoid biosynthesis pathways were highly up-regulated. Since seedlings rely on seed storage reserves (carbohydrates and lipids) for early growth, the concerted activation of fatty acid catabolism in *Osdrp1c* likely reflects an altered utilization of these reserves due to its retarded growth. Similarly, the full activation of the phenylpropanoid pathway indicates a heightened stress response (**Supplementary Figures 8f, h**), which indicates that *Osdrp1c* makes efforts to combat stress *in vivo*. Phenylpropanoids are known for their protective roles in plants, which encompass antioxidant, anti-inflammatory, and antiviral functions (Song, D. W., et al., 2021). Besides, oxidative phosphorylation is the primary source of ROS; thus, up-regulation of V-type ATPases in in this pathway (**Supplementary Figure 8b**)—reflecting enhanced ATP synthesis—is inevitably accompanied by ROS generation. To maintain redox homeostasis, genes involved in the glutathione metabolism pathway (**Supplementary Figures 8c, h**)—central to ROS scavenging—are substantially upregulated. Additionally, other pathways, including ascorbate and aldarate metabolism (**Supplementary Figures 8d, h**), flavonoid biosynthesis (**Supplementary Figure 8e**), and the phenylpropanoid pathway (**Supplementary Figures 8f, h**) (which generates non-enzymatic antioxidants), are also activated to participate in ROS scavenging. Within the ribosome pathway, 51 genes were significantly down-regulated versus only 4 up-regulated (**Supplementary Figure 8g**), indicating a severe impairment of protein translation capacity in *Osdrp1c*.

Dynamic expression pattern of ROS homeostasis genes in *Osdrp1c* compared with WT was analyzed. Among all ROS homeostasis genes detected in the RNA-seq datasets, expression of genes involved in ROS production were followed: AO (Alternative oxidase) (1 induced and 0 repressed), Rboh (1 induced and 0 repressed), SOD (1 induced and 0 repressed) (**Figures 9e, f**); Genes involved in ROS production were consistently and significantly induced (**Figure 9f**). Expression of genes involved in ROS scavenging were followed: GR (Glutathione reductase) (1 induced and 0 repressed), APX (1 induced and 2 repressed), CAT (3 induced and 0 repressed), TRX (Thioredoxin) (3 induced and 7 repressed), Prx (peroxidase) (39 induced and 14 repressed), GST (35 induced and 5 repressed) (**Figures 9e, g**); Most genes involved in ROS scavenging were significantly induced (**Figure 9g**), which further indicated that ROS were accumulated in *Osdrp1c*, plant initiates ROS scavenging pathway and decreases excess ROS in order to maintain ROS homeostasis.

### 3.6 Metabolite profiles of *Osdrp1c*

**Figure 10:**
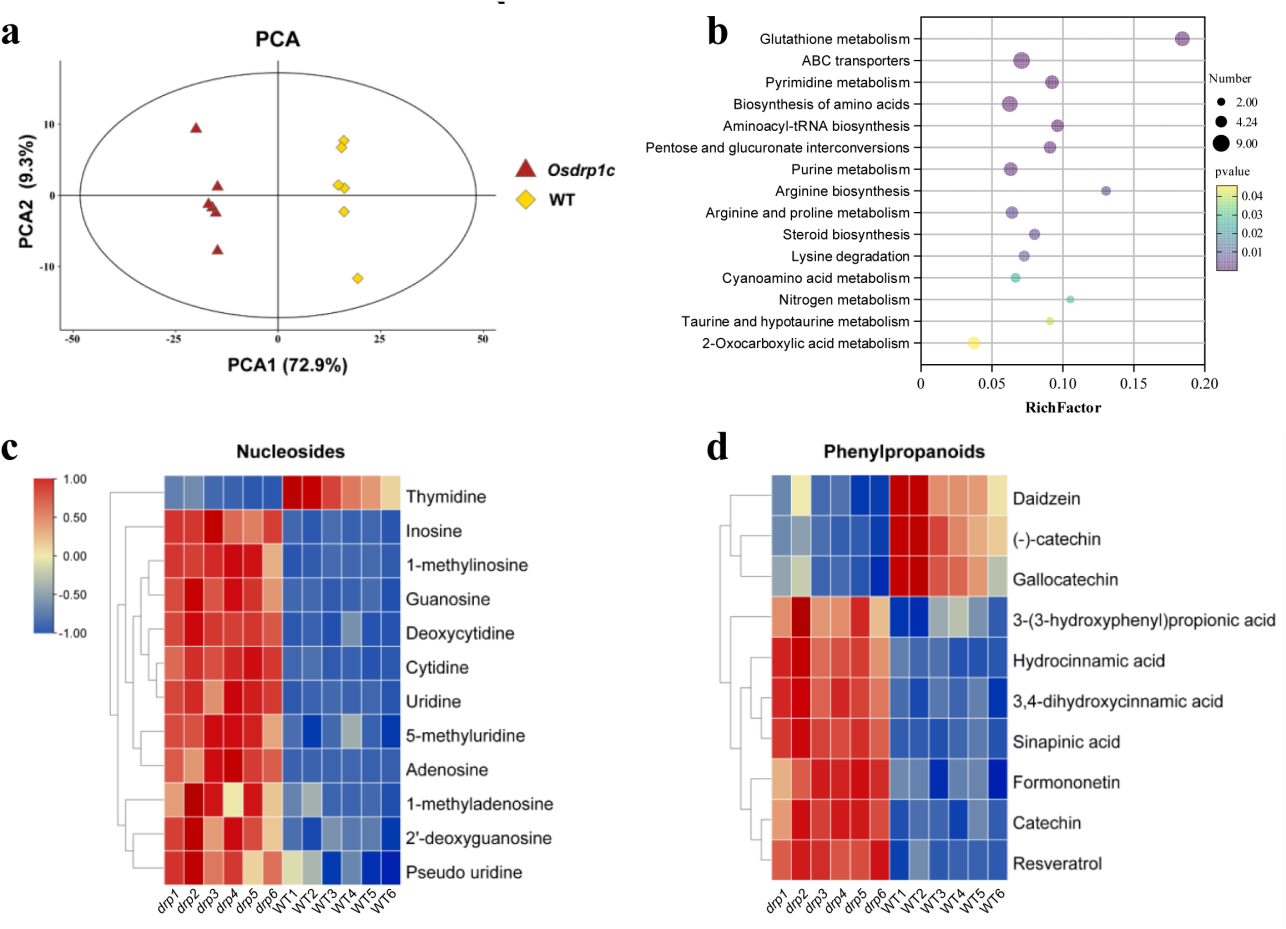
Metabolomic analysis of roots of *Osdrp1c* by GC-MS. **(a)** PCA plot of all samples, including the QC sample. **(b)** Enrichment analysis of KEGG metabolic pathways for the differential metabolites between *Osdrp1c* and WT, *P* < 0.05. **(c, d)** Metabolites involved in nucleosides biosynthesis **(c)** and phenylpropanoid biosynthesis **(d)** were significantly different for *Osdrp1c. drp1*~*6*, 6 biological replicates of *Osdrp1c* roots samples, WT1~6, 6 biological replicates of WT roots samples.

Non-targeted GC-MS metabolomics revealed extensive metabolic reprogramming in *Osdrp1c* roots. Univariate analysis identified 337 differential metabolites (*P* < 0.05), with a strong bias toward up-regulation (281 up vs. 56 down). Supervised multivariate analysis (PLS-DA/OPLS-DA) using VIP>1 and *P* < 0.05 further refined this to 256 robust DEMs (226 increased, 30 decreased). The stability of the platform was confirmed by PCA of QC samples (**Figure 10a**). The severe root growth phenotype of *Osdrp1c*, coupled with this widespread metabolic disruption, indicates that root development is tightly coupled with specific metabolic states.

To explore the metabolic pathways associated with OsDRP1C function, we performed KEGG enrichment analysis on the differential metabolites. The ten most significantly enriched pathways were primarily associated with: (1) redox balance and transport (glutathione metabolism, ABC transporters); (2) genetic material synthesis and translation (pyrimidine and purine metabolism, aminoacyl-tRNA biosynthesis); (3) amino acid metabolism (arginine, proline, and lysine biosynthesis); and (4) specialized metabolism (pentose and glucuronate interconversions, steroid biosynthesis) (**Figure 10b**). Enrichment analysis of KEGG GSH metabolic pathways (**Supplementary Figure 9a**), GSH, responsible for scavenging excess ROS to maintaining redox homeostasis (Fedoreyeva and Kononenko, 2025), is an up-regulated metabolite. This result is consistent with the previous transcriptomic data (**Supplementary Figure 8b**). The coordinated increase of tyrosine, tryptophan, and methionine, alongside the depletion of glutamate and accumulation of glycine (**Supplementary Figure 9b**), points to a systemic metabolic reprogramming toward enhanced glutathione biosynthesis and phenylpropanoid-mediated ROS scavenging. This pattern aligns with established multi-omics evidence in rice under oxidative stress (Song, Y. et al., 2021; Ghate et al., 2022; Jan et al., 2023). The concurrent elevation of 2-oxoglutarate (**Supplementary Figure 9b**) suggests increased TCA cycle flux to meet the energy demand for stress repair. These results demonstrate that OsDRP1C significantly reprograms the metabolic profile of rice roots, with pronounced impacts on pathways central to ROS scavenging. To further characterize the altered metabolites, we conducted annotation by integrating information from the KEGG, HMDB, and METLIN databases. This analysis revealed that nucleic acids (**Figure 10c**) and secondary metabolites (phenylpropanoids) (**Figure 10d**) were significantly different for *Osdrp1c*.

## 4 Discussion

### 4.1 The unique role of *OsDRP1C* among known root development genes

Similar to auxin-related mutants (e.g., *AUX1*, *PIN2*) (Justamante et al., 2025), the *Osdrp1c* mutant exhibits severely impaired primary and lateral root growth, albeit through a distinct mechanism. *OsDRP1C*, encoding a dynamin-related protein, maybe indirectly modulates auxin distribution by affecting vesicle trafficking and the subcellular localization of PIN proteins, rather than directly transporting auxin itself.

In contrast to the *WOX11* mutant, which shows reduced adventitious root number (Geng et al., 2023), *Osdrp1c* displays increased adventitious root number but with shortened length. This suggests a negative regulatory role for *OsDRP1C* in adventitious root initiation and a positive role in their subsequent elongation, indicating a more complex functional repertoire.

At the cellular level, root elongation of all types (primary, adventitious, lateral) is inhibited in *Osdrp1c*, resembling the effect of *Rht-D1b* (Wang et al., 2025). However, the increased adventitious root number is a unique phenotype, potentially an indirect consequence of altered root-shoot ratio or local hormone (e.g., auxin) redistribution.

### 4.2 Conservation and divergence of OsDRP1C within the plant DRP family

In this study, we identified a rice short-root mutant, *Osdrp1c*, exhibiting defects in cell elongation, division, endocytosis, and increased ROS accumulation. OsDRP1C encodes a plasma membrane-localized dynamin-related protein (DRP). Comparative analysis with *Arabidopsis* homologs reveals both functional conservation and species-specific divergence. In *Arabidopsis*, DRP1C localizes to the cell plate and root hair tips, with loss-of-function causing male gametophyte lethality (Konopka et al., 2008). However, its root function was uncharacterized—a gap addressed by this study. Notably, the *Arabidopsis drp1a* mutant (*rsw9*) displays strikingly similar root phenotypes to *Osdrp1c*: shortened roots, reduced cell elongation, and decreased endocytosis (Collings et al., 2008). This suggests that in *Arabidopsis*, DRP1A assumes root developmental roles performed by DRP1C in rice, while AtDRP1C specializes in reproduction, indicating subfunctionalization along species-specific trajectories. Our *Osdrp1c* allele (T-to-A mutation at 2858 bp, premature stop codon) differs from two previously reported alleles (Kawai et al., 2022), which may explain phenotypic discrepancies.

Plant DRP isoforms exhibit localization-dependent functional specialization. Rice OsDRP2B, required for cell wall biogenesis, loss causes short roots and brittle culm with reduced cellulose (Hirano et al., 2010). OsDRP1E, a mitochondrial DRP, suppresses PCD by maintaining cristae integrity; its mutation causes cytochrome C release and cell death (Li et al., 2017). Similarly, *Arabidopsis* DRP3A/3B mediate mitochondrial and peroxisomal fission (Fujimoto et al., 2009), while DRP5B (ARC5) is involved in chloroplast, peroxisome, and mitochondrial division (Gao et al., 2003; Zhang and Hu, 2010; Aung and Hu, 2012). OsDRP1C-GFP localizes to the chloroplast, cytoplasm and plasma membrane (**Figure 8k**), suggesting a role in chloroplast and cell division. We propose that aberrant DRP1C in *Osdrp1c* disrupts cell division and expansion through impaired dynamin function.

### 4.3 DRP1C, endocytosis, and cell wall synthesis: A mechanistic pathway for root growth regulation

Reduced endocytic activity in *Osdrp1c* aligns with phenotypes in *Arabidopsis drp1a* (Collings et al., 2008) and rice Osdrp1c (Kawai et al., 2022). In mammals, dynamin mediates clathrin-mediated endocytosis (CME) via vesicle scission. Although plant DRP1s lack PH/PR domains, DRP1C-GFP co-localizes with clathrin at dynamic cortical foci in *Arabidopsis*, with foci dynamics dependent on functional CME (Konopka et al., 2008). This provides mechanistic insight: endocytic cycling of plasma membrane (PM) proteins—including receptors, transporters, and cellulose synthase (CESA) complexes—regulates cell polarity and wall synthesis. The *drp1a* mutant exhibits reduced cellulose content, altered polysaccharide composition, and hypersensitivity to the endocytosis inhibitor monensin (Collings et al., 2008), suggesting that DRP1A deficiency disrupts CESA complex cycling between PM and intracellular compartments, impairing cellulose deposition and cell elongation. Extending this model, OsDRP1C likely maintains cell wall integrity by regulating endocytic trafficking of CESA complexes or associated proteins. The reduced elongation and division in Osdrp1c can be explained by a causal chain: endocytic defects→disrupted CESA trafficking→compromised wall integrity→impaired elongation. Given DRP1C enrichment at the cell plate (Collings et al., 2008), this directly correlates with aberrant division phenotypes. Thus, OsDRP1C regulates root development primarily through endocytosis-mediated PM composition and wall deposition dynamics.

### 4.4 ROS signaling: coupling endocytic defects with oxidative stress

*Osdrp1c* roots show increased ROS accumulation and cell death. Two non-mutually exclusive explanations exist: (1) impaired cell wall integrity activates wall surveillance pathways, triggering ROS as a stress signal; (2) DRP1C loss directly or indirectly affects ROS homeostasis genes. Our multi-omics data support the latter: OsDRP1C modulates GSH and phenylpropanoid metabolism, with significant flux perturbations in related pathways. This connects DRP1C to redox regulation. During rice crown root development, auxin signaling regulates antioxidant gene expression (peroxidases, glutathione reductases, GSTs) to maintain ROS homeostasis for organogenesis (Kumar et al., 2024). Disrupted GSH pathways in *Osdrp1c* may represent a molecular interface between endocytic dysfunction and redox imbalance. A plausible hypothesis: OsDRP1C regulates endocytic cycling of PM transporters for antioxidant precursors (glutamate, cysteine, glycine); its loss alters transporter abundance/localization, impairing GSH biosynthesis, reducing ROS scavenging, and causing oxidative damage. Notably, this differs from animal systems, where Drp1 mediates mitochondrial fission and directly regulates mitochondrial ROS production (Jiang et al., 2022) (Jiang et al., 2022) and ferroptosis (Clemente, 2022). *Arabidopsis* DRP1C does not co-localize with mitochondria (Konopka et al., 2008), indicating plant DRP1C indirectly affects ROS via PM endocytosis, wall integrity, and metabolic reprogramming—a species-specific adaptation to sessile lifestyles requiring cell wall integrity as a core adaptive structure.

### 4.5 Metabolic hub of OsDRP1C regulatory network: GSH and phenylpropanoid pathways

Our metabolomic data reveal extensive perturbations in GSH, amino acid, nucleotide, and phenylpropanoid pathways in *Osdrp1c*. GSH is a primary antioxidant involved in cell cycle regulation and PCD; phenylpropanoids generate lignin monomers and flavonoids essential for wall mechanics, development, and stress responses. Coordinated dysregulation of these pathways suggests OsDRP1C influences multiple metabolic networks through upstream nodes. Integrated transcriptomic and metabolomic analyses generate hypotheses: Does OsDRP1C regulate PM localization of transcription factors or signaling receptors via endocytosis, controlling redox-related gene expression? Is glutathione precursor transport dependent on DRP1C-mediated endocytosis? Do PM-localized phenylalanine transporters or RLKs require such regulation? Answering these questions will advance DRP1C studies from single-gene correlations toward systemic network dissection.

### 4.6 Working model and future directions

We propose a working model: As a PM-localized dynamin-related GTPase, OsDRP1C participates in CME, regulating PM abundance and distribution of proteins including CESA complexes, nutrient/antioxidant metabolite transporters, and receptor kinases. This endocytic function: (1) ensures normal wall synthesis and elongation by maintaining CESA trafficking; (2) sustains redox homeostasis by modulating GSH metabolism-related transporters or signaling components; (3) when lost, impaired endocytosis causes wall defects and metabolic dysregulation, with ROS accumulation triggering cell death and inhibiting root elongation.

Study limitations include: (1) direct substrates/interactors of OsDRP1C unidentified—CME involvement inferred from Arabidopsis homologs (Konopka et al., 2008) requires verification in rice; (2) molecular link between decreased endocytosis and GSH dysregulation unclear—indirect stress response or specific “antioxidant metabolite transporters” as direct targets?; (3) functions in other tissues (especially reproductive organs) need characterization—contrast between Arabidopsis drp1c lethality and viable rice mutant suggests differential functional compensation mechanisms between monocots and dicots.

Future directions: (1) screen interactors via IP-MS; (2) tissue-specific knockout/overexpression combined with cell biology (FM4-64, cellulose staining, ROS localization); (3) validate metabolomic perturbations by examining GSH synthesis enzymes (γ-ECS, GS) and exogenous GSH/precursor rescue; (4) investigate functional differentiation between rice OsDRP1C and *Arabidopsis* DRP1A/1C via phylogenetics and complementation. These studies will deepen understanding of DRP functions in crop development and identify potential targets for root architecture improvement.

## Data Availability

The datasets presented in this study can be found in online repositories. The names of the repository/repositories and accession number(s) can be found in the article/**Supplementary Material**.

## Supplementary material

Supplementary Figure 1 Quantification of adventitious roots number in WT and *Osdrp1c* from 1 week to 5 weeks. Significant differences were determined using Student’s test (**P* < 0.05, ***P* < 0.01, ****P* < 0.001).

Supplementary Figure 2 Schematic representation of mutation sites in the *Osdrp1c* (-1, -9, -15).

Supplementary Figure 3 Cross sections of the root maturation zone (top), elongation zone (middle) and meristem zone (bottom) of 3-day-old WT and *Osdrp1c* plants. Bar 100 μm.

Supplementary Figure 4 Quantification of grain length and width of the WT and *Osdrp1c* (Rough rice (with husk) and brown rice (dehulled)). Significant differences were determined using Student’s test (**P* < 0.05, ***P* < 0.01, ****P* < 0.001).

Supplementary Figure 5 Identification of transcriptional levels of genes in ROS production and scavenging pathways of 7-day-old WT and *Osdrp1c* leaves.

Supplementary Figure 6 NBT staining of RAMs of 7-day-old WT and *Osdrp1c* after being cheated with DMTU or DPI. Bar 1 mm.

Supplementary Figure 7 Semi qRT -PCR of WT, *Osdrp1c*, comp 1 and comp 2.

Supplementary Figure 8 Transcriptomic analysis focused on ROS-mediated redox homeostasis of roots of 7-day-old *Osdrp1c* by RNA-seq. **(a)** Volcano plot of the global distribution of DEGs, non-significant genes are depicted in gray, significantly up-regulated (red) and down-regulated (green) genes are as indicated. Boxes labelled with red, green, and yellow indicate up-regulated, down-regulated, and both up- and down-regulated DEGs between WT and *Osdrp1c*. Mapping of DEGs associated with the KEGG oxidative phosphorylation **(b),** glutathione metabolism **(c)**, ascorbate and aldarate metabolism **(d),** flavonoid biosynthesis **(e)** and phenylpropanoid pathway **(f)**. **(g)** MapMan analysis of DEGs associated with ribosome proteins. **(h)** Heatmap of DEGs in redox homeostasis-related genes; red represents highly expressed genes, beige indicates moderately expressed genes, and blue denotes lowly expressed genes. Log2FoldChange ≥1.0, and ≤-1.0 with Padj<0.05 was considered as up-regulated and down-regulated, respectively in **(a, h)**. DHAR (Dehydroascorbate reductase), GRX (Glutaredoxin), CCR (Cinnamoyl-CoA reductase), IRL (Isoflavone reductase-like protein), DREB (Dehydration-responsive element-binding protein), PAL (Phenylalanine ammonia-lyase), CHS (Chalcone synthase), FLS/F3H (Flavonol synthase/Flavanone 3-hydroxylase).

Supplementary Figure 9 Metabolomic analysis of roots of *Osdrp1c* by GC-MS. **(a, b)** Enrichment analysis of KEGG GSH metabolic pathways **(a)** and biosynthesis of amino acids **(b)** for the differential metabolites between *Osdrp1c* and WT. In metabolic pathway diagrams, small circles represent metabolites. Metabolites highlighted in red are experimentally detected as up-regulated, while those in blue are down-regulated.

Supplementary Table 1 Primers used in the experiments.
