# Supplementary figures and images for: The dynamin-related protein Osdrp1c plays an essential role in rice root elongation (Oryza sativa L.)
Source: Front Plant Sci. 2026 Apr 2;17:1783261. doi: 10.3389/fpls.2026.1783261 (PMC13083138; doi:10.3389/fpls.2026.1783261)

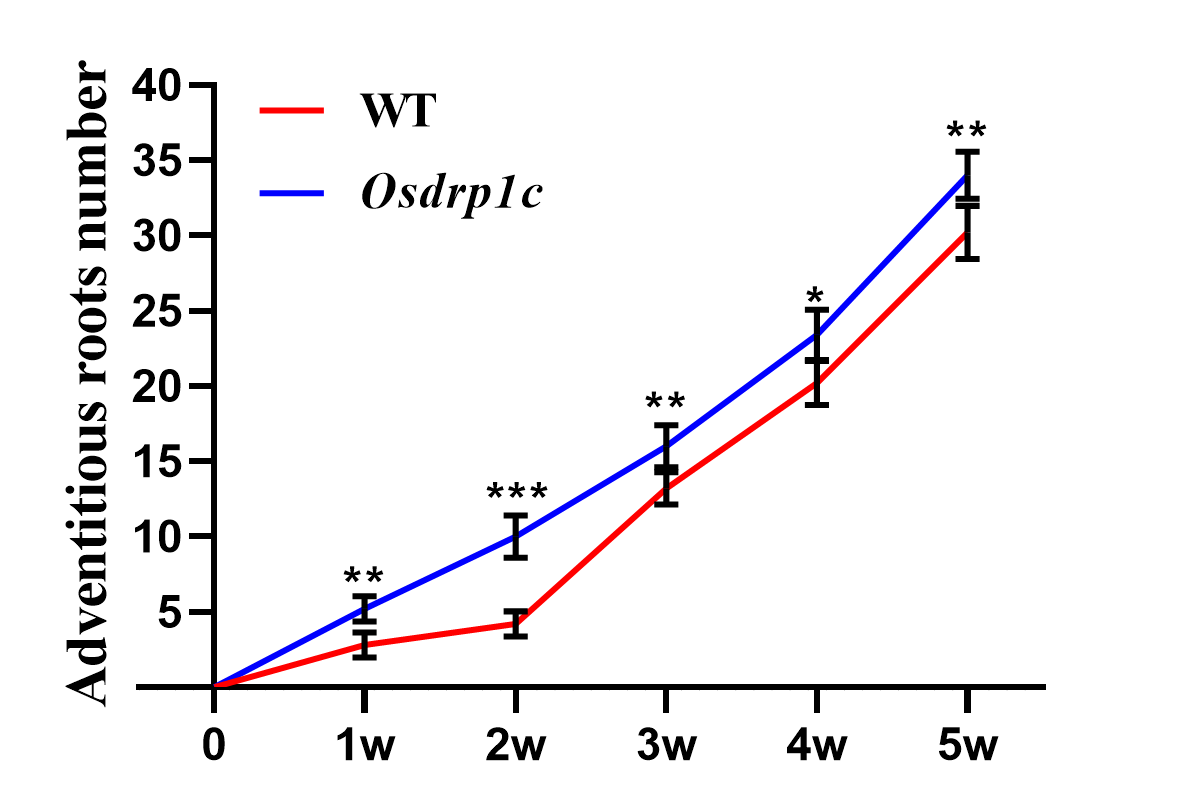

Supplement: Supplementary Figure 1 — Quantification of adventitious roots number in WT and Osdrp1c from 1 week to 5 weeks. Significant differences were determined using Student’s test (*P < 0.05, **P < 0.01, ***P < 0.001). [file Image1.tif]

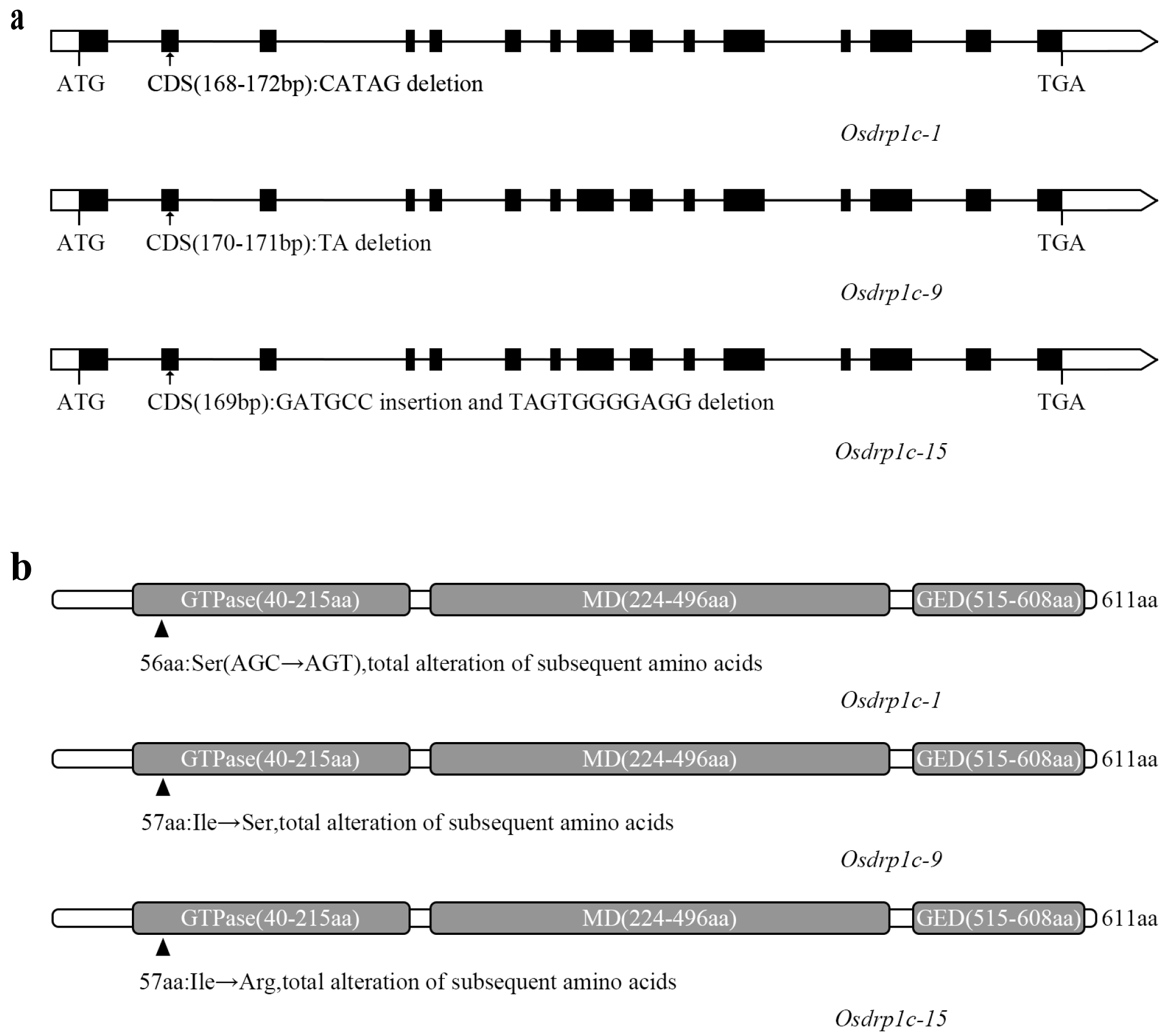

Supplement: Supplementary Figure 2 — Schematic representation of mutation sites in the Osdrp1c (-1, -9, -15). [file Image2.tif]

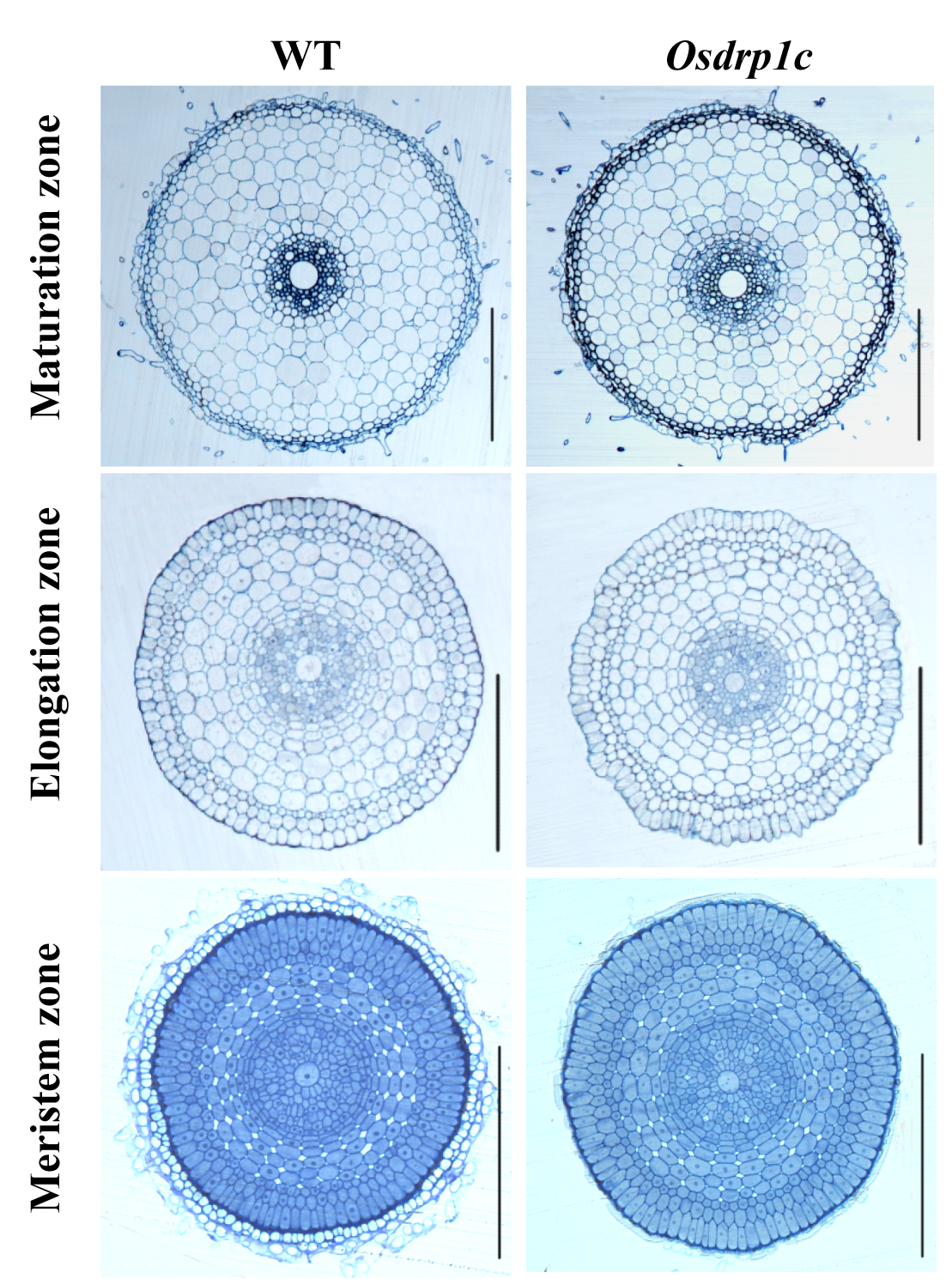

Supplement: Supplementary Figure 3 — Cross sections of the root maturation zone (top), elongation zone (middle) and meristem zone (bottom) of 3-day-old WT and Osdrp1c plants. Bar 100 μm. [file Image3.tif]

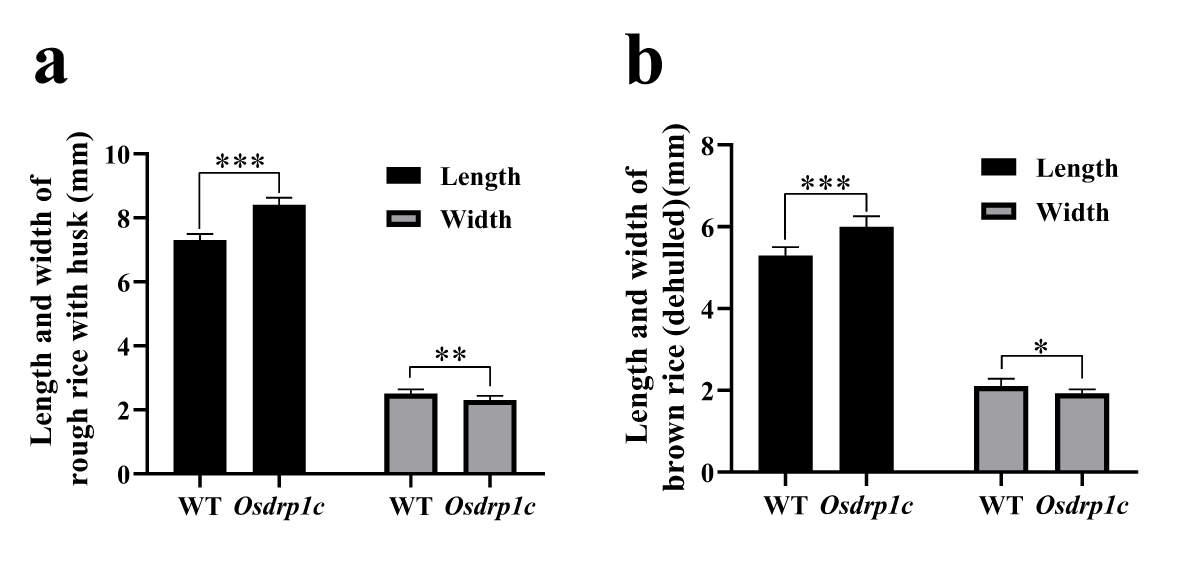

Supplement: Supplementary Figure 4 — Quantification of grain length and width of the WT and Osdrp1c (Rough rice (with husk) and brown rice (dehulled)). Significant differences were determined using Student’s test (*P < 0.05, **P < 0.01, ***P < 0.001). [file Image4.tif]

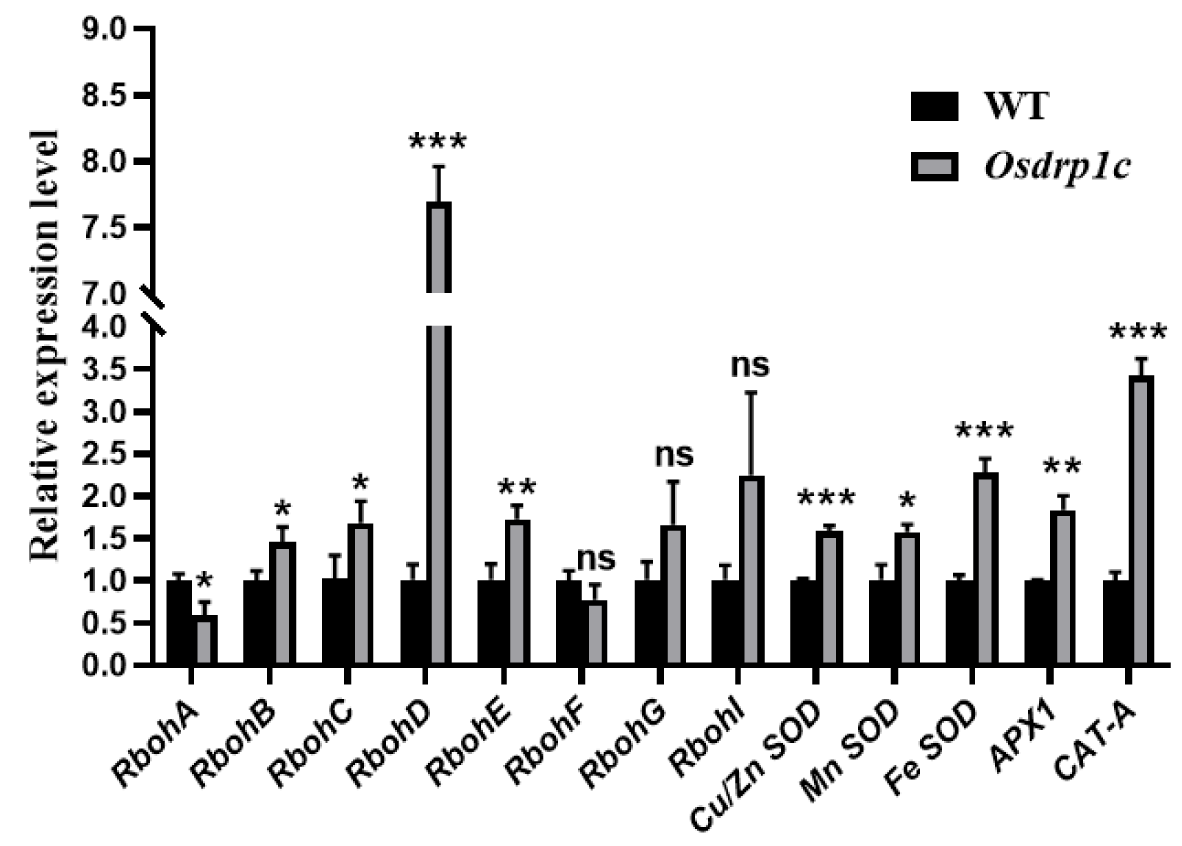

Supplement: Supplementary Figure 5 — Identification of transcriptional levels of genes in ROS production and scavenging pathways of 7-day-old WT and Osdrp1c leaves. [file Image5.tif]

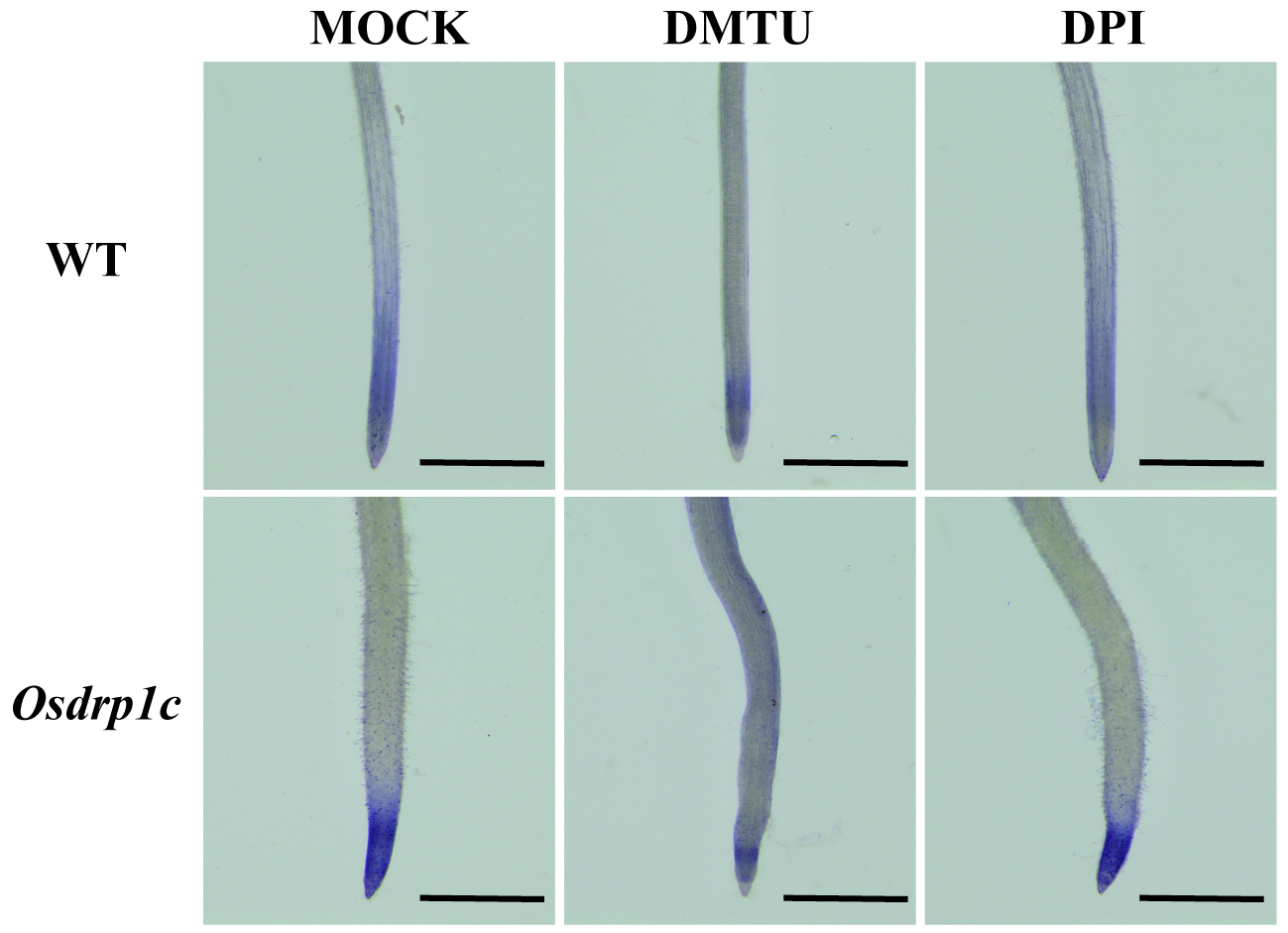

Supplement: Supplementary Figure 6 — NBT staining of RAMs of 7-day-old WT and Osdrp1c after being cheated with DMTU or DPI. Bar 1 mm. [file Image6.tif]

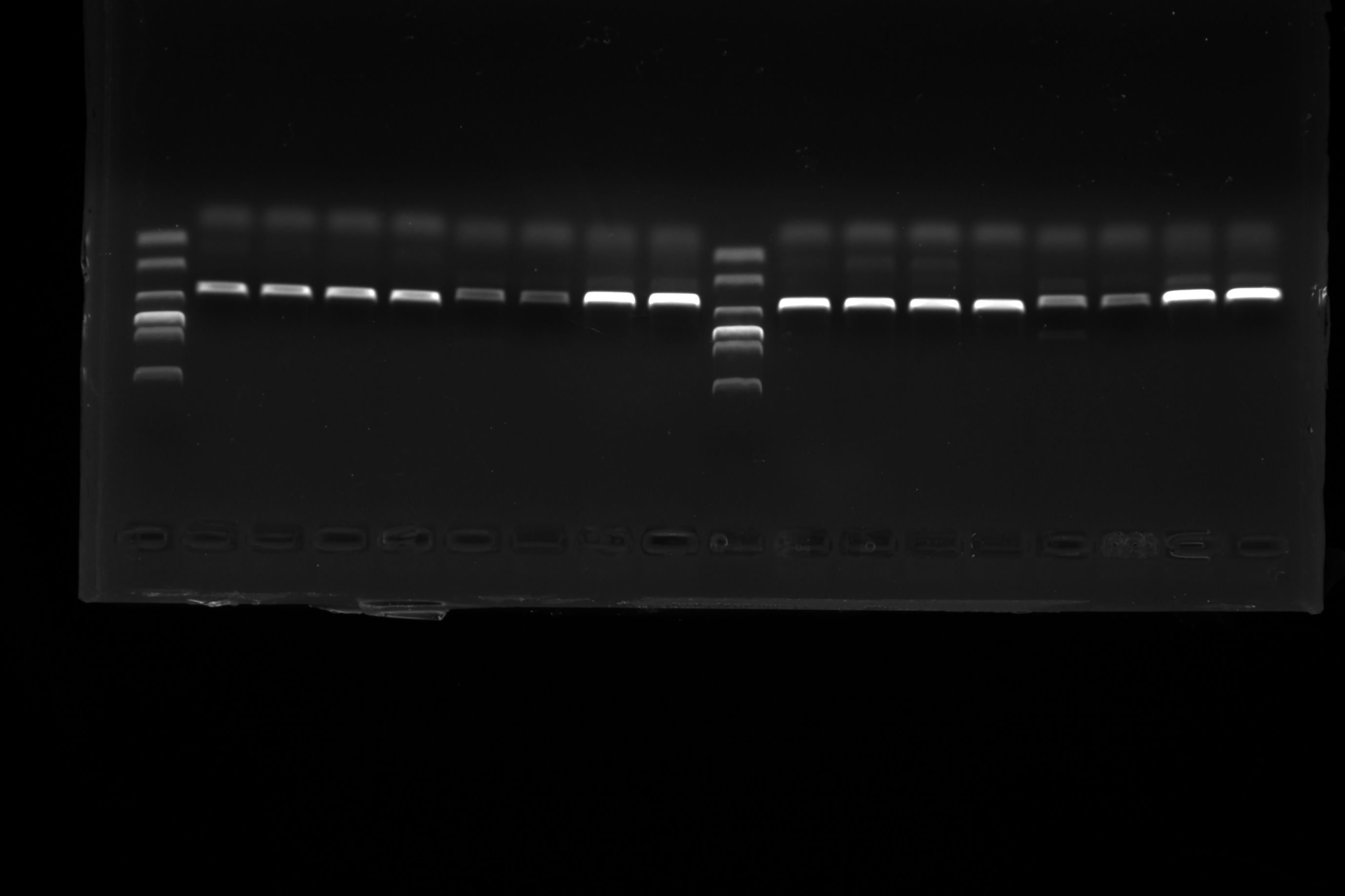

Supplement: Supplementary Figure 7 — Semi qRT -PCR of WT, Osdrp1c, comp 1 and comp 2. [file Image7.jpeg]

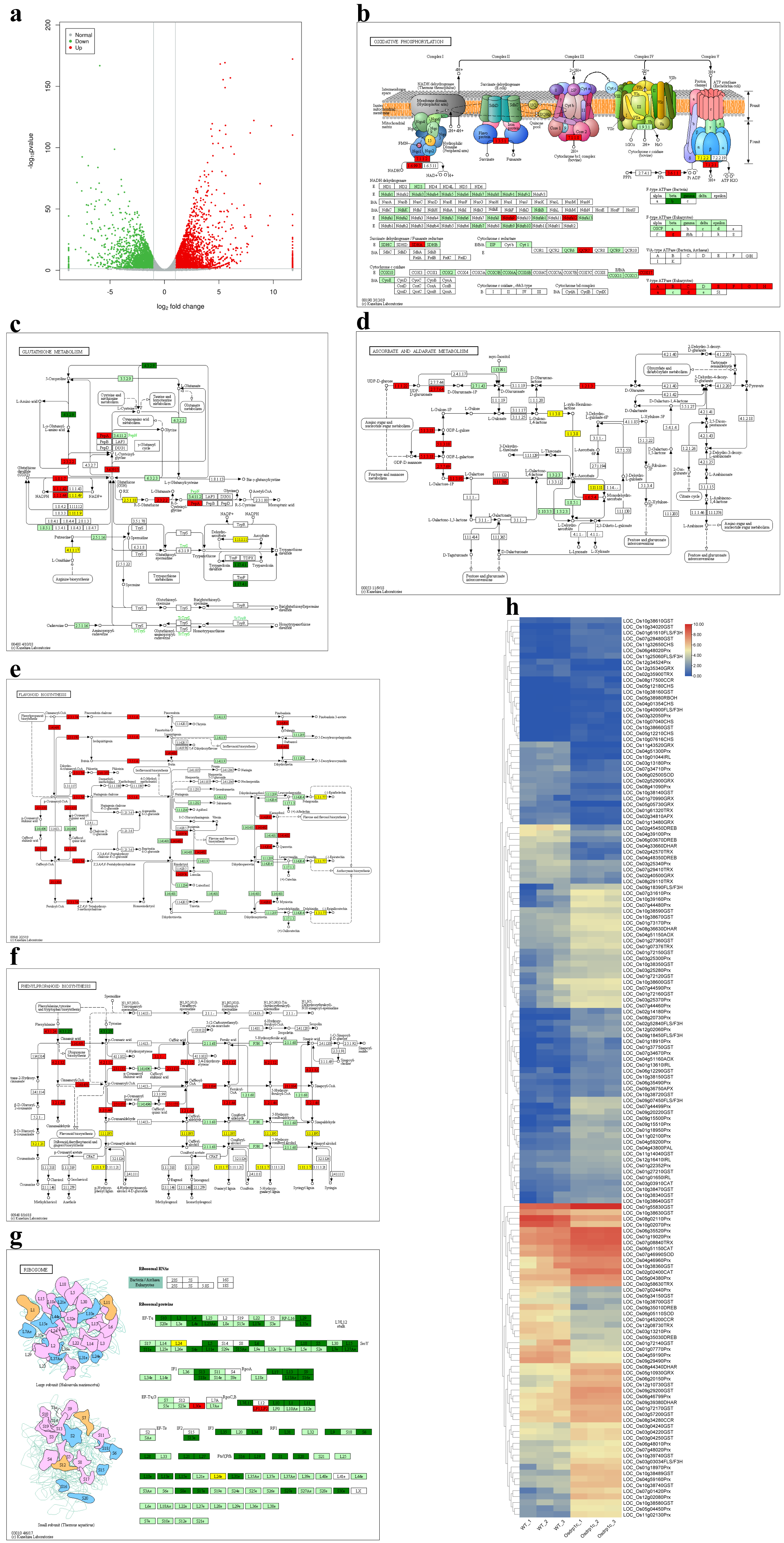

Supplement: Supplementary Figure 8 — Transcriptomic analysis focused on ROS-mediated redox homeostasis of roots of 7-day-old Osdrp1c by RNA-seq. (a) Volcano plot of the global distribution of DEGs, non-significant genes are depicted in gray, significantly up-regulated (red) and down-regulated (green) genes are as indicated. Boxes labelled with red, green, and yellow indicate up-regulated, down-regulated, and both up- and down-regulated DEGs between WT and Osdrp1c. Mapping of DEGs associated with the KEGG oxidative phosphorylation (b), glutathione metabolism (c), ascorbate and aldarate metabolism (d), flavonoid biosynthesis (e) and phenylpropanoid pathway (f). (g) MapMan analysis of DEGs associated with ribosome proteins. (h) Heatmap of DEGs in redox homeostasis-related genes; red represents highly expressed genes, beige indicates moderately expressed genes, and blue denotes lowly expressed genes. Log2FoldChange ≥1.0, and ≤-1.0 with Padj<0.05 was considered as up-regulated and down-regulated, respectively in (a, h). DHAR (Dehydroascorbate reductase), GRX (Glutaredoxin), CCR (Cinnamoyl-CoA reductase), IRL (Isoflavone reductase-like protein), DREB (Dehydration-responsive element-binding protein), PAL (Phenylalanine ammonia-lyase), CHS (Chalcone synthase), FLS/F3H (Flavonol synthase/Flavanone 3-hydroxylase). [file Image8.tif]

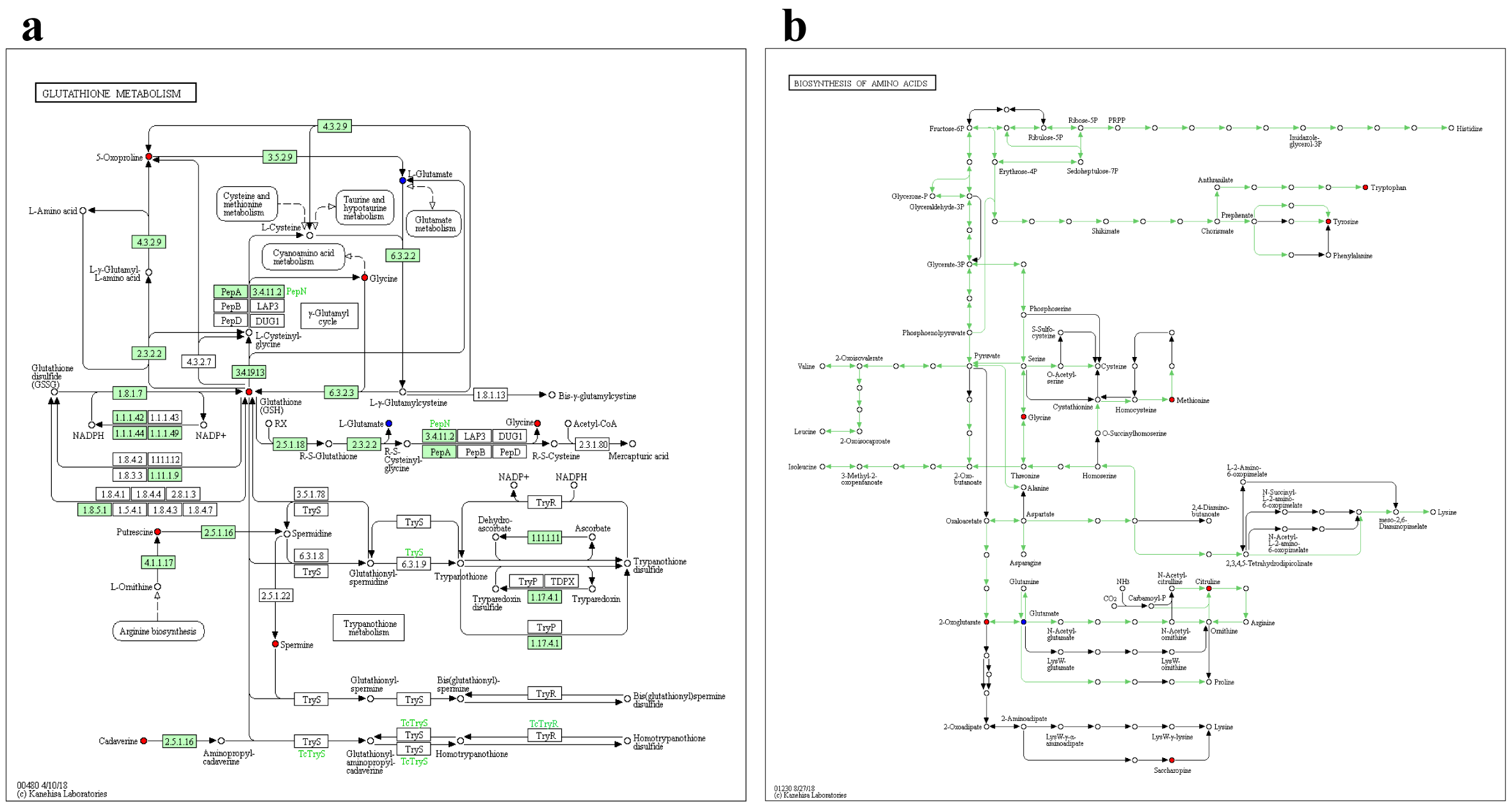

Supplement: Supplementary Figure 9 — Metabolomic analysis of roots of Osdrp1c by GC-MS. (a, b) Enrichment analysis of KEGG GSH metabolic pathways (a) and biosynthesis of amino acids (b) for the differential metabolites between Osdrp1c and WT. In metabolic pathway diagrams, small circles represent metabolites. Metabolites highlighted in red are experimentally detected as up-regulated, while those in blue are down-regulated. [file Image9.tif]
